# Evidence for Anion‐Anion Interaction in Amino Acid Ionic Liquids Probed by Far‐Infrared Spectroscopy

**DOI:** 10.1002/cphc.202500297

**Published:** 2025-06-30

**Authors:** David Kotwica, Ralf Ludwig

**Affiliations:** ^1^ Institut für Chemie Abteilung für Physikalische Chemie Universität Rostock Albert‐Einstein‐Str. 27 18059 Rostock Germany; ^2^ Department LL&M University of Rostock Albert‐Einstein‐Str. 25 18059 Rostock Germany; ^3^ Leibniz‐Institut für Katalyse e.V. Albert‐Einstein‐Str. 29a 18059 Rostock Germany

**Keywords:** anionic dimers, DFT calculations, enthalpies of vaporization, far infrared, ionic liquids

## Abstract

Herein, it is shown that far‐infrared spectroscopy is a sensitive probe for detecting not only cation‐anion but also anion‐anion interaction in imidazolium‐based ionic liquids (ILs). The low‐frequency spectra of ILs with the same 1‐ethyl‐3‐methylimidazolium cation but different anions are measured, namely the acetate and glycinate anion, whereby the latter forms the simplest amino acid IL (AAIL). It is shown that two glycinate anions form two hydrogen bonds between either the carboxylate and amino groups allowing for attractive anion‐anion interaction despite the Coulomb repulsion between the ions of like charge. The spectral signature of the torsional motion of the amine groups ranges between 200 and 350 cm^−1^, strongly depending on the binding motif as determined by cation‐anion or anion‐anion interactions. This is confirmed by density functional theory calculations of frequencies of larger IL clusters exhibiting these characteristic binding motifs. The spectral signatures assigned to the cation‐anion interactions are correlated to measured enthalpies of vaporization, confirming a once proposed relation which can be obviously used for predicting thermodynamic properties from spectroscopy for less stable AAILs.

## Graphical abstract

Spectroscopic evidence for doubly hydrogen bonded anionic dimers in amino acid ionic liquids is provided. The torsional motion of the amine groups is characteristic for cation‐anion and anion‐anion interaction as observed in far‐infrared spectra.

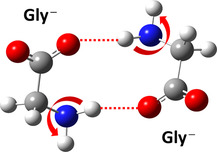

## 1 Introduction

Ionic liquids (ILs) present a new liquid material with potential application in science and technology.^[^ ^1^, ^2^, ^3^, ^4^, ^5^, ^6^, ^7^ ^]^ ILs are formed solely by ions and reveal ubiquitous properties, such as a broad liquid range, low vapor pressure, and high thermal stability. Their characteristics allow various applications as electrolytes in batteries or in solar cell as well as solvents in chemical processes. The structure, dynamics, and thermodynamics of ILs strongly depend on the delicate mélange of Coulomb interaction, hydrogen bonding, and dispersion forces between the IL constituents: cations and anions. There are indirect probes for describing the cation‐anion interaction such as the NMR proton chemical shifts δ^1^H or the IR stretch frequencies ν(C‐H) at the ring protons in imidazolium‐based ILs.^[^ ^8^, ^9^, ^10^, ^11^, ^12^, ^13^, ^14^, ^15^, ^16^, ^17^, ^18^, ^19^, ^20^, ^21^ ^]^ For the direct measure of this interaction strength between cation and anion, several spectroscopic methods provide access to the low‐frequency range from 10 to 600 cm^−1^ or 0.3 to 15 THz. The palette of suitable methods includes optical heterodyne‐detected Raman‐induced Kerr‐effect spectroscopy,^[^ ^22^, ^23^, ^24^, ^25^, ^26^, ^27^, ^28^, ^29^, ^30^, ^31^ ^]^ terahertz (THz) spectroscopy,^[^ ^31^, ^32^, ^33^, ^34^, ^35^, ^36^ ^]^ dielectric relaxation spectroscopy,^[^ ^31^, ^37^, ^38^ ^]^ low‐energy neutron scattering,^[^ ^39^ ^]^ far‐infrared (FIR) spectroscopy,^[^ ^40^, ^41^, ^42^, ^43^, ^44^, ^45^, ^46^ ^]^ as well as Raman spectroscopy.^[^ ^47^, ^48^, ^49^, ^50^ ^]^ Recently, Hamm et al. added 2D Raman‐THz spectroscopy for studying imidazolium‐based ILs in the low‐frequency range below 5 THz.^[^ ^51^ ^]^

In this study, we measured the FIR spectra of the IL 1‐ethyl‐3‐methylimidazolium acetate [EMIm][OAc] and the amino acid ILs (AAIL) [EMIm][Gly] between 30 and 500 cm^−1^ for a broad temperature range between 193 and 353 K. In a pioneering work, Ohno et al. synthesized AAILs in a systematic way using 1‐ethyl‐3‐methylimidazolium ([EMIm]^+^) as cation and 20 natural amino acids as anions.^[^ ^52^ ^]^ AAILs have the advantageous properties to be biodegradable, nontoxic, and inexpensive. Moreover, their physicochemical properties can easily be adjusted for a wide range of applications.

**Scheme 1:**
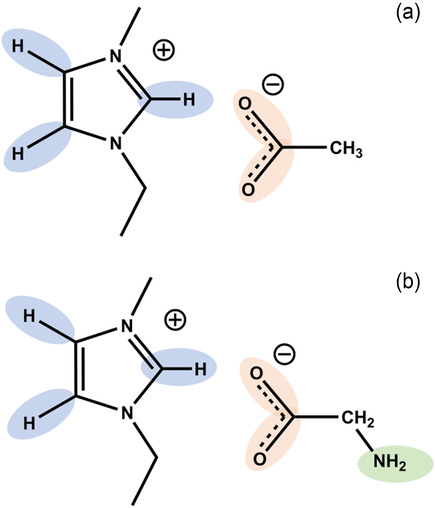
We investigated a) the IL 1‐ethyl‐3‐methylimidazolium acetate [EMIm][OAc] and b) the amino acid IL (AAIL) 1‐ethyl‐3‐methylimidazolium glycinate [EMIm][Gly]. They both have the proton donor abilities at the imidazolium cation (blue) and the acceptor abilities at the carboxylate group of the anions (red). They differ in the functional amine group (green) which is only present in the AAIL.

Here, we used the simplest AAIL [EMIm][Gly] as model compound in comparison to the IL [EMIm][OAc] for a systematic study of the low‐frequency motions of cations, anions, and ion pairs being characteristic for the binding motifs. They comprise the same 1‐ethyl‐3‐methylimidazolium cation but different anions. Both, the acetate anion OAc^‐^ and the glycinate anion Gly^‐^ exhibit proton accepting carboxylate groups which are known to form Coulomb‐enhanced hydrogen bond (H‐bonds) with the acidic C(2)—H position between the two nitrogen atoms of the 1‐ethyl‐3‐methylimidazolium as observed in the FIR spectra around 140 cm^−1^.^[^ ^53^ ^]^ As also shown in **Scheme** **1**, the glycinate anion in the AAIL additionally provides an amine group NH_2_, allowing for potential proton donor‐acceptor interaction and therefore the formation of doubly H‐bonded anionic dimers as reported in quantum chemical calculations of AAIL clusters.^[^ ^54^ ^]^ First experimental evidence for hydrogen bonds between the amine hydrogens and the carboxylate group of other anions has been observed in pair correlation functions derived from neutron diffraction.^[^ ^55^ ^]^ Recently, the formation of anionic clusters due to hydrogen bonding has been also reported from molecular dynamics (MD) simulations of cholinium‐based AAILs. The simulations showed that the additional peptide group from [Ch][Pep] adds a new anchoring point for interanionic association through H‐bonding.^[^ ^56^ ^]^ For similar ILs Kirchner et al. reported distinct cation‐anion, cation‐cation, as well as anion‐anion interactions observed in radial pair and combined distribution functions from MD simulations.^[^ ^57^ ^]^ In this respect, there is little experimental evidence for the existence of anionic dimers in AAILs.

Thus, choosing [EMIm][OAc] and [EMIm][Gly] as model compounds allows a reasonable comparison of the H‐bonds between cation and anion in ordinary ion pairs with the unusual ones between ions of the same charge, in our case anionic dimers. The assignment of the spectral signatures of the cation‐anion and the anion‐anion interaction will be realized by comparison with frequencies obtained from DFT calculations explicitly including dispersion forces on larger IL clusters up to pentamers.^[^ ^58^, ^59^, ^60^, ^61^ ^]^ Additional Natural Bond Orbital (NBO) calculations show that the second order stabilization energies are good descriptors for spectroscopic properties such vibrational frequencies and NMR proton chemical shifts.^[^ ^62^, ^63^ ^]^ For both ILs, we found that the low frequency vibrational bands and enthalpies of vaporization are correlated and provide a path for estimating thermodynamic properties from spectroscopic measurements.

## 2 Experimental Section

### 2.1 Materials and Sample Preparation

The IL [EMIm][OAc] and the AAIL [EMIm][Gly]) were both purchased from IoLiTec (Ionic Liquids Technologies GmbH, Heilbronn) with a purity of 96%. We characterized the ILs by standard liquid NMR and IR spectroscopy. The AAIL [EMIm][Gly] was characterized by elemental analysis because a Karl‐Fischer titration was not applicable. For the IL [EMIm][OAc] we measured a water concentration of 150 ppm.

Each sample was dried thoroughly using a high‐vacuum membrane pump at a pressure of 2 × 10^−5^ mbar at a temperature of 60 °C for at least one day. Upon drying, the probes were stored under nitrogen atmosphere. Prior to each measurement, the probes were dried again for at least 1 h. Afterward, the ILs were filled in a standard liquid cuvette from SPECAC. As the window material, PE windows with a 100 μm (spacer) were chosen. Within the filling process, the samples were taken into a syringe and filled into the cell under nitrogen stream to avoid contact with the atmosphere.^[^ ^46^ ^]^

### 2.2 Far‐Infrared Spectroscopy

The FIR measurements were performed with a Bruker VERTEX 70 FTIR spectrometer. The instrument was equipped with an extension for measurements in the FIR region. This equipment consisted of a multilayer mylar beam splitter, a room‐temperature DLATGS detector with preamplifier, and polyethylene (PE) windows for the internal optical path. The accessible spectral region for this configuration lay between 30 and 680 cm^−1^. Further improvement could be achieved using a high‐pressure mercury lamp and a silica beam splitter. The reason why mercury lamps have proved to be so successful for FIR is that the emission from the plasma reinforces the emission from the hot quartz envelope of the lamp. This configuration allowed measurements down to 10 cm^−1^ and significantly had better signal‐to‐noise ratios compared to the above given configuration. Background spectra were recorded with an empty cell. In all cases, the sample temperature was maintained by an external Haake DC 30/K 20 bath chiller and recorded with a NiCrNi thermocouple attached directly to the cell.^[^ ^40^, ^41^, ^46^ ^]^

### 2.3 DFT Calculations

We calculated clusters of the ILs [EMIm][OAc] and [EMIm][Gly] consisting of varying number of ion pairs *n *= 1 − 5 (see Supporting Information). For that purpose, we employed B3LYP‐D3/6‐31+G* calculations performed with the Gaussian 09 program and analyzed with the NBO 6.0 program.^[^ ^62^, ^63^ ^]^ For calculating all clusters at the same level of theory, we used the well‐balanced 6‐31+G* Pople basis set. Including polarization as well as diffuse functions, this basis set is suitable for reasonably calculation of hydrogen‐bonded clusters in ILs. We demonstrated that the salient properties of these clusters can be robustly calculated with both smaller and larger basis sets so long Grimme's D3 dispersion correction is considered.^[^ ^59^, ^60^, ^61^ ^]^ For all geometry‐optimized clusters, we calculated the frequencies, since they are sensitive probes for hydrogen bonding. These IR spectroscopic observables are related to NBO calculated second order stabilization energy Δ*E* ^(2)^ *n* → σ*.^[^ ^62^, ^63^ ^]^

## 3 Results and Discussion

**Figure 1:**
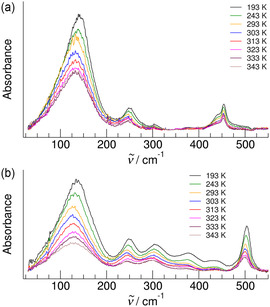
Far‐infrared spectra between 30 and 550 cm^−1^ of a) the IL [EMIm][OAc] and b) the AAIL [EMIm][Gly] measured as a function of temperature ranging from 193 up to 343 K.

We recorded the FIR spectra of the IL 1‐ethyl‐3‐methylimidazolium acetate [EMIm][OAc] and the amino acid IL (AAIL) 1‐ethyl‐3‐methylimidazolium glycinate [EMIm][Gly] in the frequency range from 30 to 550 cm^−1^ as shown in **Figure** **1**. Because ILs provide a broad liquid range, we measured the low‐frequency spectra at temperatures between 193 and 343 K, covering a Δ*T* of about 150 K. Two peculiarities in the FIR spectra are immediately apparent and can be only related to the additional amino group in the glycinate anion of the AAIL [EMIm][Gly]. First, the broad vibrational band between 30 and 200 cm^−1^ which is usually assigned to the bending and stretching modes of cation‐anion (c‐a) interaction, is wider for [EMIm][Gly] than for [EMIm][OAc]. Obviously, new vibrational modes are added in the frequency range up to 100 cm^−1^ related to (c‐a) interaction with the amino group. Secondly, the frequency range between 200 and 500 cm^−1^ is more populated for [EMIm][Gly], probably due to intramolecular bending and torsional modes of the NH_2_ groups.

**Figure 2:**
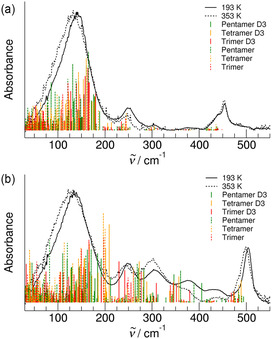
Far‐infrared spectra of a) the IL [EMIm][OAc] and b) the AAIL [EMIm][Gly] shown for the lowest temperature at 193 K (full line) and the highest temperature at 353 K (dotted line). The DFT‐calculated frequencies of the corresponding IL clusters including three, four, and five ion pairs are shown for comparison. The full and the dotted peaks show the vibrational frequencies obtained from calculation with and without taking dispersion interaction explicitly into account by using Grimme's D3 approach^[^ ^59^, ^60^, ^61^ ^]^ considering dispersion interaction leads to blueshifts in the FIR spectra due to stronger interaction.

To verify and deepen our assumptions, we performed DFT calculations of clusters of both ILs with up to five ion pairs (*n *= 1 − 5). The calculations were performed with and without explicit consideration of the dispersion interaction by using Grimme's D3 approach.^[^ ^59^, ^60^, ^61^ ^]^ All vibrational frequencies are plotted against the measured spectra recorded at the lowest and highest temperatures as shown in **Figure** **2**. Given the limitation that the frequencies were calculated in harmonic approximation, the measured spectral signatures are well reproduced. As expected, the vibrational contributions calculated with dispersion interaction are observed at higher frequencies, which is due to the overall stronger interaction. The calculated frequencies allowed us to assign the vibrational band present in the FIR spectra of both ILs.

**Figure 3:**
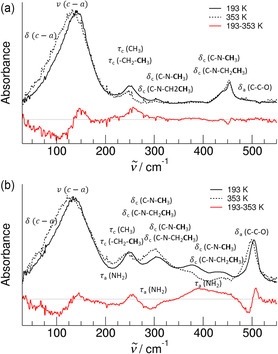
Assignment of the far‐infrared spectra of a) the IL [EMIm][OAc] and b) the AAIL [EMIm][Gly]. The frequencies below 200 cm^−1^ can be assigned to the bending mode δ(c‐a) and the stretching mode ν(c‐a) of the cation‐anion interaction related to the ^+^C(2)‐H^**…**^OOC^‐^ hydrogen bond. For AAIL, we observe increasing and decreasing intensities we assign to the torsional motion of the amine groups *τ*_a_(NH_2_) depending on the structural motifs (see Figure 4 in more detail). The red curves indicate the differences between the spectra recorded at 193 and 353 K, respectively.

For [EMIm][Gly], we find the bending and stretching motions *δ *(c‐a) and ν (c‐a) with a maximum at 136 cm^−1^ at 193 K which is shifted to lower wavenumbers with increasing temperature caused by weaker interaction (see **Figure** **3**). The vibrational bands at 250 cm^−1^ are assigned to torsional motions *τ* of the methyl and ethyl groups and those between 300 and 430 cm^−1^ to the bending modes *δ*(C—N—CH_3_) and *δ*(C—N—CH_2_—CH_3_) of the imidazolium cation. The bending modes *δ*(C—C—O) are observed for the acetate anion. With the exception of the vibrational modes associated with the (c‐a) interaction, no significant temperature dependence is observed, indicating that the vibrational modes originate from weak intramolecular motions that change only slightly with temperature.

**Figure 4:**
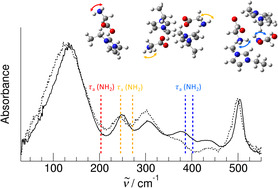
Assignment of the far‐infrared spectra of the AAIL [EMIm][Gly] focusing on the torsional motion of the amine groups *τ*_a_(NH_2_) depending on wherein they are involved the structural motifs. The contribution around 400 cm^−1^ can be clearly assigned to the torsional motion *τ*_a_(NH_2_) of amino groups involved in anionic dimers formed by two strong hydrogen bonds ^+^NH^**…**^OOC^‐^ (blue dotted peaks and arrows).

The vibrational features of the IL [EMIm][OAc] are also observed in the AAIL [EMIm][Gly] because the AAIL includes the same imidazolium cation and the same carboxylate group at the anion. The additional bands between 200 and 400 cm^−1^ in the spectra of [EMIm][Gly] are assigned to the torsional motion *τ*_a_(NH_2_) of the amine group. These characteristic bands are strongly shifted depending on which way the amine groups are bound within the H‐bonding motifs. As shown in **Figure** **4**, the torsional modes *τ*_a_(NH_2_) at around 200 cm^−1^ belong to an amine group that rotates almost free if cation and anion are strongly hydrogen bonded via ^+^C(2)—H^…^OOC^‐^. This band is shifted about 60 cm^−1^ to higher frequencies if this type of (c‐a) ion pair experiences the neighborhood of another similar bound ion pair mimicking the overall environment in the IL. However, the contribution around 400 cm^−1^ is clearly assignable to the torsional motion *τ*_a_(NH_2_) of amino groups involved in anionic dimers formed by two strong hydrogen bonds ^+^NH^…^OOC^‐^, obviously overcoming the repulsive Coulomb forces between the like charged ions. Thus, we provide the first spectroscopic evidence for doubly H‐bonded anionic dimers in ILs as predicted before in quantum chemical calculations on AAIL clusters and observed in pair correlation functions from MD simulations and from neutron diffraction of ILs including amino acid anions.^[^ ^54^, ^55^, ^56^, ^57^, ^58^ ^]^ Our assignment of the three different types of torsional motion *τ*_a_(NH_2_) of amino groups is supported by the observed spectral changes with temperature. The hydrogen bonds forming the anion dimer (a‐a) are weaker than the (c‐a) hydrogen bonds. Therefore, the vibrational band at 400 cm^−1^ decreases with temperature for the benefit of increasing contributions for the torsional band *τ*_a_(NH_2_) of between 200 cm^−1^ and 300 cm^−1^. Entropic effects may also play a role, as the anions in the doubly hydrogen‐bonded dimers have fewer degrees of freedom. Thus, the anionic dimers are mainly present at low temperatures as reflected in the difference spectrum obtained from subtracting the spectrum recorded at the highest temperature 353 K from that measured at the lowest temperature 193 K. As shown in Figure 3b, the band of the torsional mode *τ*_a_(NH_2_) at around 400 cm^−1^ exhibits positive intensities, whereas those at around 200 and 300 cm^−1^ are negative.

**Figure 5:**
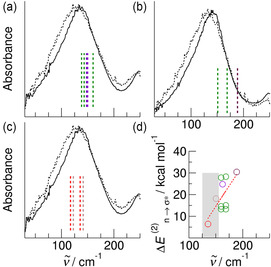
Intermolecular motion between cation and anion (c‐a) observed in the FIR spectra of a) the AAIL [EMIm][Gly] and b) the IL [EMIm][OAc]. Intermolecular motion between anion and anion (a‐a) is only observed for [EMIm][Gly] at lower frequencies as shown in c). It is clearly observed that the (a‐a) contributions are responsible for the lower frequencies resulting in a less steeper decrease of the flank of the broad band (see red and brownish dotted peaks). In d) the NBO‐calculated second‐order stabilization energy Δ*E* ^(2)^ *n* → σ* plotted versus the calculated frequencies for the (c‐a) and (a‐a) interactions. The data pairs describing the (a‐a) interaction lie in the gray area characterized by lower frequencies and smaller stabilization energies.

**Figure 6:**
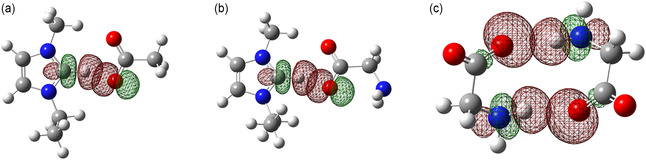
NBO‐calculated second order stabilization energy Δ*E* ^(2)^ *n* → σ* visualized for the types of interactions observed in the FIR spectra. a,b) Overlap between the lone pair orbitals *n* of one of the carboxylate oxygens and the antibond orbitals σ* of the C(2)—H bond in [EMIm][OAc] and [EMIm][Gly]. c) Overlap between the lone pair orbitals *n* of one of the carboxylate oxygens and the antibond orbitals σ* of the N—H bonds as present in the anionic dimers of [EMIm][Gly]. As expected, the H‐bonds in ion pairs (c‐a) are stronger than those in the anionic dimers (a‐a).

More evidence for our interpretation of the spectra is provided by the low‐frequency range below 200 cm^−1^. The broad vibrational band with a maximum at 136 cm^−1^ in the spectra of [EMIm][OAc] results from the stretching motion between cation and anion along the ^+^C(2)—H^…^OOC^‐^ H‐bonds. This band is even broader in the spectra of [EMIm][Gly] due to the anion‐anion interaction along the two ^+^NH^…^OOC^‐^ H‐bonds. In **Figure** **5** we show the low‐frequency spectra of the AAIL [EMIm][Gly] with the calculated spectral signatures for the (c‐a) and the (a‐a) hydrogen bonds. In average, the (a‐a) frequencies are redshifted by 20 cm^−1^ compared to the (c‐a) frequencies which are also observed in the [EMIm][OAc] spectra (see Figure 5a,c, and b). Plotting the calculated frequencies for the (c‐a) and (a‐a) H‐bonds versus the calculated second order stabilization energy Δ*E* ^(2)^ *n* → σ* from NBO analysis, we obtain an almost linear behavior as shown in Figure 5d. This supports that the NBO parameters are reasonable descriptors for spectroscopic observables including IR frequencies.^[^ ^62^, ^63^ ^]^ In **Figure** **6** **,** we visualize the second order stabilization energy Δ*E* ^(2)^ *n* → σ* indicating the strength of the hydrogen bonds for three typical structural motifs. The overlap between the lone pair orbitals *n* of one of the carboxylate oxygens and the antibond orbitals σ* of the C(2)—H bond in [EMIm][OAc] and [EMIm][Gly] is shown in Figure 6a,b, whereas the overlap between the lone pair orbitals *n* of one of the carboxylate oxygens and the antibond orbitals σ* of the N—H bonds as present in the anionic dimers of [EMIm][Gly] is visualized in Figure 6c. The second order stabilization energy Δ*E* ^(2)^ *n* → σ* of the anionic dimer is obviously smaller supporting the observation of lower wavenumbers in the FIR spectra assigned to this cluster species (see also Figure 5d).

**Figure 7:**
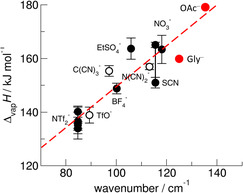
Measured maxima of the low‐frequency vibrational bands from FIR plotted versus enthalpies of vaporizations for a set of imidazolium‐based ILs including differently strong interacting anion (see ref. [44]). Here, we added the data for the IL [EMIm][OAc] derived from surface tension measurement and for the AAIL [EMIm][Ala] calculated from MD simulations. The latter should be similar to that of [EMIm][Gly].^[^ ^65^, ^66^ ^]^

We should emphasize that the resulting wavenumbers for the vibrational modes are determined by the force constants as well as by the reduced masses via $\tilde{\nu }=\frac{1}{2\pi c}\sqrt{\frac{k}{\mu }}$, where *c* is the speed of light, *k* the force constant and *μ* the reduced mass. However, FIR spectra of protic ILs (PILs) combined with DFT calculations on PIL clusters clearly showed that half of the shifts to lower wavenumbers are determined by decreasing force constants and half by increasing reduced masses.^[^ ^64^ ^]^ Overall, we could show that the frequency shifts describe the weakening or strengthening of the interaction between the ions in ILs. Some time ago, this gave us the idea of relating the low‐frequency (c‐a) vibration bands to enthalpies of vaporization for imidazolium‐based ILs. Rather than relating transport properties and thermodynamic properties, it seemed more reliable to focus on properties that describe the interaction energies between the constituents of ILs and can quantify the energetics involved in the vaporization process.^[^ ^24^, ^25^, ^26^, ^27^ ^]^ Low enthalpies of vaporization corresponded to low wavenumbers and higher Δ_vap_ *H* values could be referred to higher frequencies *ν*(c‐a). For a set of imidazolium‐based ILs a linear relationship was proposed between the low‐frequencies *ν*(c‐a) and measured enthalpies of vaporization. In **Figure** **7** **,** we added our measured frequencies of [EMIm][OAc] and [EMIm][Gly] and plotted them against enthalpies of vaporization of the IL [EMIm][OAc] obtained from surface tension measurements and the AAIL [EMIm][Ala] (which should be similar to [EMIm][Gly]) calculated from MD simulations.^[^ ^54^, ^65^, ^66^ ^]^ Our results confirm the correlation between the spectroscopic and thermodynamic properties and show that such a relation may open a reasonable path for predicting enthalpies of vaporization for thermally less stable ILs.^[^ ^67^, ^68^ ^]^

## 4 Conclusion

In conclusion, we show that far‐infrared (FIR) spectroscopy is a suitable method for describing the interaction strength between cation and anion in the IL [EMIm][OAc] and the AAIL [EMIm][Gly]. For the simplest AAIL [EMIm][Gly], we could even observe anionic dimers which have been predicted by quantum chemical calculations and MD simulations in the literature. In this cluster species, two glycinate anions form two hydrogen bonds between either the carboxylate and amino groups. We could show that attractive anion‐anion interaction is possible via H‐bonding despite the Coulomb repulsion between the ions of like charge. The spectral signature for proofing the existent anion dimers is not the weaker (a‐a) interaction compared to that of the usual (c‐a) interaction, but the torsional motion of the amine groups in the glycinate anion. The NH_2_ torsional band ranges between 200 and 400 cm^−1^, strongly depending on the binding motif as determined by cation‐anion or anion‐anion interactions. This is confirmed by density functional theory (DFT) calculations of frequencies on larger IL clusters exhibiting these characteristic binding motifs. The contributions of the intermolecular (a‐a) interaction is buried under the low frequency flank of the broad vibrational band between 30 and 200 cm^−1^ and is about 20 cm^−1^ redshifted compared to that of the (c‐a) interaction. That the (a‐a) H‐bonds are weaker than the (c‐a) H‐bonds is supported by calculated NBO second order stabilization energies. Finally, we could show that the spectral signatures assigned to interionic interactions are strongly correlated to enthalpies of vaporization, conforming a relation once proposed for predicting thermodynamic properties from spectroscopy.

## Conflict of Interest

The authors declare no conflict of interest.

## Supporting information

Supplementary Material

## Data Availability

The data that support the findings of this study are available in the supplementary material of this article.
