# Supplementary material for: Evidence for Anion‐Anion Interaction in Amino Acid Ionic Liquids Probed by Far‐Infrared Spectroscopy
Source: Chemphyschem. 2025 Jun 30;26(19):e202500297. doi: 10.1002/cphc.202500297 (PMC12503908; doi:10.1002/cphc.202500297)
Supplement: Supplementary file 1 — Supplementary Material [file CPHC-26-e202500297-s001.pdf]

Evidence for Anion-Anion Interaction in Amino Acid Ionic Liquids probed by Far-Infrared Spectroscopy

David Kotwica[a], Ralf Ludwig[a,b,c]*

a Universität Rostock, Institut für Chemie, Abteilung für Physikalische Chemie, Albert-Einstein-Str. 27, 18059 Rostock, Germany; Tel: 49 381 498 6517; E-mail: ralf.ludwig@uni-rostock.de

b Department LL&M, University of Rostock, Albert-Einstein-Str. 25, 18059 Rostock, Germany

c Leibniz-Institut für Katalyse an der Universität Rostock e.V. Albert-Einstein-Str. 29a, 18059 Rostock, Germany

Supporting Information (SI)

Table of contents:

- FIR parameters
- Sample preparation
- Computational details
- Calculated Structures

FIR parameters:

The FTIR measurements were performed with a Bruker Vertex 70 FTIR spectrometer equipped with an extension for measurements in the FIR region that consists a multilayer mylar beam splitter, a room temperature DLATGS detector with preamplifier, and polyethylene (PE) windows for the internal optical path. The accessible spectral region for this configuration lies between 30 and 680 cm^{-1} (0.3 and 20,3 THz). Further improvement could be achieved by using a high pressure mercury lamp and a silica beam splitter. This configuration allowed measurements down to 10 cm^{-1} (0.1 THz) and significantly better signal-to-noise ratios.

Sample preparation:

Both ionic liquids have been purchased from Iolitec and have been characterized by standard liquid NMR spectroscopy and IR spectroscopy. The 1-Ethyl-3-methylimidazolium glycinate has further been characterized by elemental analysis.

Each sample have been dried thoroughly by a high vacuum membrane pump at a pressure of $2*10^{-5}$ mbar at a temperature of 60 °C for at least one day. Upon drying the samples were stored under nitrogen atmosphere. Prior each measurement the samples were dried again for at least one hour. Afterwards the ILs were filled in a standard liquid cell from SPECAC. As window material PE windows with a 100 µm (spacer) has been chosen. Within the filling process the probes have been taken into a syringe and filled into the cell under nitrogen stream to avoid contact with the atmosphere. The cell was sealed with screws and was put into the spectrometer within a temperature control module (Variable Temperature Cell Holder, SPECAC).

Temperature control was ensured by a thermostat which was in embedded in a bore of the cell. Heating was ensured by a thermocouple and cooling was achieved by adding liquid nitrogen into the bore of the mount.

Computational details:

All computations of the corresponding oligomers have been conducted with the B3LYP method and the 6-31+G* basis set. All optimization steps have been carried out prior the final optimization and frequency calculations. For the utilized structures all convergence criteria have been met (Maximum force, RMS force, Maximum Displacement, RMS displacement). Calculation of each oligomer highlighted in this work were conducted either with empirical dispersion interaction (GD3BJ from the Grimme group) or without.

Calculated Structures:

	EMIm OAc with D3	EMIm OAc without D3	EMIm Gly with D3	EMIm Gly without D3
Monomer	1 a)	1 b)	1 c)	1 d)
Dimer	2 a)	2 b)	2 c)	2 d)
Trimer	3 a)	3 b)	3 c)	3 d)
Tetramer	4 a)	4 b)	4 c)	4 d)
Pentamer	5 a)	5 b)	5 c)	5 d)
Dimer (Double H-Bonded)			2 e)	

1 a) XYZ-coordinates of calculated oligomers:

EMIM Acetate structures in xyz-coordinates:

1 a)

1-Ethyl-3-methylimidazolium acetate Monomer with dispersion interactions:

C	2.73505500	-0.25198500	-0.02238100
O	1.81809600	-1.12124500	-0.21234200
O	2.53324200	0.97934700	0.15996300
C	-0.56742700	0.31690700	-0.13367100
C	-2.79089100	0.38287000	-0.24109800
C	-2.34064800	1.65258600	-0.02126100
N	-0.95672700	1.58645600	0.04285800
H	0.46965300	-0.11256200	-0.14042500
H	-3.79127700	-0.00424000	-0.35674500
H	-2.87384500	2.58425600	0.08825200
N	-1.66886400	-0.42798900	-0.31110900
C	-1.62928500	-1.88936800	-0.48905700
C	-2.05763500	-2.62596600	0.77735700
H	-0.59707400	-2.13299400	-0.75195000
H	-2.27540900	-2.13606200	-1.33740400
H	-2.00992600	-3.70629100	0.60496400
H	-1.38592300	-2.38216600	1.60628100
H	-3.08329400	-2.37129400	1.06764400
C	-0.03026900	2.70584500	0.26098500
H	-0.25553300	3.17465200	1.22247000
H	0.98491700	2.28973500	0.25999200
H	-0.15100000	3.43380600	-0.54560700
C	4.17525200	-0.76901800	-0.02160000
H	4.88590700	0.04149500	0.15955200
H	4.29020600	-1.54018000	0.74940800
H	4.39613100	-1.24347700	-0.98529400

2 a)

1-Ethyl-3-methylimidazolium acetate Dimer with dispersion interactions:

C	-1.78112100	-2.09117900	0.94421700
O	-0.88011800	-2.34820000	0.08533300
O	-1.78526600	-1.09066800	1.71750400
C	1.38369400	-0.71743400	0.98370100
C	3.51352900	-0.11370800	0.84252500
C	2.93112300	0.62169900	1.83248600
N	1.60990600	0.22038300	1.91018700
H	0.43290700	-1.21855700	0.75476300
H	4.51492600	-0.09506600	0.44493100
H	3.32633400	1.40292000	2.46194400
N	2.53326500	-0.94952800	0.33824800
C	2.64144600	-1.81465300	-0.84404000
C	3.77573400	-2.82590000	-0.71678300
H	2.76525400	-1.14794900	-1.70250700
H	1.67575700	-2.31677100	-0.93354200
H	3.79903200	-3.45807600	-1.61078400
H	4.75260900	-2.33675400	-0.63258700
H	3.63373200	-3.47065500	0.15705500
C	0.56917100	0.83948400	2.73005200

H	0.42167900	1.85884000	2.36453300
H	-0.34306600	0.25017000	2.60434400
H	0.88622900	0.84198100	3.77626000
C	-2.93773200	-3.09065200	1.06325100
H	-3.83586400	-2.60829600	1.46106000
H	-3.15133900	-3.56083900	0.09807700
H	-2.64577300	-3.88580900	1.76152000
C	-1.38369300	0.71743100	-0.98370000
C	-3.51352800	0.11370500	-0.84253300
C	-2.93111900	-0.62170000	-1.83249400
N	-1.60990100	-0.22038300	-1.91019000
H	-0.43290700	1.21855200	-0.75475800
H	-4.51492600	0.09506200	-0.44494300
H	-3.32632800	-1.40291800	-2.46195600
N	-2.53326600	0.94952300	-0.33825000
C	-2.64145000	1.81464700	0.84403800
C	-3.77573800	2.82589400	0.71677800
H	-1.67576200	2.31676500	0.93354300
H	-2.76526100	1.14794300	1.70250400
H	-3.79903800	3.45807100	1.61077900
H	-3.63373200	3.47065000	-0.15705900
H	-4.75261200	2.33674900	0.63257800
C	-0.56916300	-0.83948200	-2.73005200
H	-0.88621700	-0.84197500	-3.77626200
H	0.34307400	-0.25016900	-2.60433900
H	-0.42167300	-1.85883900	-2.36453700
C	1.78111900	2.09118400	-0.94421100
O	1.78527100	1.09067300	-1.71749900
O	0.88011200	2.34820000	-0.08532900
C	2.93772600	3.09066200	-1.06324000
H	3.15132400	3.56085400	-0.09806600
H	3.83586400	2.60830700	-1.46103900
H	2.64577000	3.88581300	-1.76151500

3 a)

1-Ethyl-3-methylimidazolium acetate <u>Trimer</u> with <u>dispersion</u> interactions:

C	-5.48904100	0.01719400	-0.52688400
O	-5.06938200	-0.77225100	-1.42944400
O	-4.74690800	0.73201700	0.21148500
C	-2.30256000	0.05721000	-1.75198600
C	-0.42510800	1.08438000	-1.14060500
C	-0.13177600	-0.23482900	-1.34072700
N	-1.31519100	-0.85119000	-1.72764100
H	-3.36972500	-0.17306300	-1.87995100
H	0.20990000	1.90784300	-0.82826900
H	0.77678900	-0.80242200	-1.15484400
N	-1.77989600	1.24088100	-1.40192400
C	-2.57216600	2.48443300	-1.31170500
C	-2.05095500	3.43227400	-0.23932600
H	-3.59086300	2.16488600	-1.07778600
H	-2.55069000	2.96495000	-2.29616300
H	-2.69819200	4.31525000	-0.21464300
H	-2.09210500	2.95748800	0.74509100
H	-1.02544700	3.76122400	-0.43186700
C	-1.46839700	-2.26605300	-2.07202400
H	-0.89016600	-2.86784000	-1.36499300

H	-2.52898600	-2.51857800	-2.02877900
H	-1.09624400	-2.43802900	-3.08654100
C	-7.00531700	0.10313200	-0.33312500
H	-7.26115100	0.69610000	0.54903700
H	-7.45713600	0.56506100	-1.21970500
H	-7.43055500	-0.90308700	-0.24639000
C	-0.81786500	-1.46283900	1.59607100
C	-2.29701800	0.18082100	1.85317500
C	-1.07571800	0.63668000	2.26323700
N	-0.17398500	-0.40113100	2.09772600
H	-0.33161100	-2.37441400	1.23207500
H	-3.25956600	0.65701800	1.72194800
H	-0.75416000	1.60137400	2.62268800
N	-2.10933300	-1.13188900	1.44835400
C	-3.16040900	-2.03879400	0.95300300
C	-4.22673300	-2.29754400	2.01181500
H	-2.65183000	-2.96245400	0.66644000
H	-3.61559700	-1.58705400	0.06913100
H	-4.96969800	-2.99142200	1.60523900
H	-3.79590100	-2.73704500	2.91857400
H	-4.74410200	-1.36943500	2.27142800
C	1.26304100	-0.35707200	2.37532300
H	1.45710600	-0.75881300	3.37477200
H	1.76259800	-0.95645200	1.60917400
H	1.59054100	0.68224800	2.30431000
C	1.81237300	-3.13462000	-0.27883300
O	2.23573600	-1.93730500	-0.27542900
O	0.65052600	-3.50414700	0.06159000
C	2.80145400	-4.22788800	-0.69851300
H	3.30571400	-4.61238900	0.19831300
H	2.28078800	-5.06785700	-1.16756800
H	3.56621200	-3.82859400	-1.37218500
C	3.62102300	0.87286300	-0.42304300
C	5.15542300	-0.61509300	-1.02031800
C	4.57428700	-0.13326200	-2.15484900
N	3.62763800	0.79697500	-1.76038500
H	2.98326200	1.51591700	0.20390500
H	5.91802600	-1.36377100	-0.88209600
H	4.73971000	-0.37257900	-3.19346000
N	4.55295800	0.03149000	0.04276100
C	4.84859800	-0.16356900	1.47134700
C	4.78286600	-1.63365000	1.87516900
H	4.11494400	0.43708800	2.01261600
H	5.83811800	0.26251300	1.67034700
H	4.88367200	-1.71384100	2.96266500
H	3.83346200	-2.07052300	1.55831700
H	5.59516800	-2.21305000	1.42275000
C	2.76660000	1.58421700	-2.64741800
H	2.26357600	2.34164700	-2.03986600
H	3.38641200	2.06074300	-3.41099900
H	2.03108400	0.92697100	-3.11731900
C	1.66342600	3.45698900	0.85193500
O	1.44917600	3.40821800	-0.39528800
O	2.16936400	2.51224300	1.53482800
C	1.24796700	4.72844200	1.59272900
H	1.42917500	5.61115900	0.97202300

H	1.77118500	4.81965100	2.54838000
H	0.16887700	4.68689000	1.79334600

4 a)

1-Ethyl-3-methylimidazolium acetate Tetramer with dispersion interactions:

C	-2.34028000	2.87906300	-1.50001600
O	-2.70207700	2.38291100	-0.38180100
O	-1.16695100	2.88530100	-1.94801900
C	-0.68593600	0.03736700	-0.57214000
C	1.52553000	-0.12768300	-0.73150400
C	1.22148600	0.23261100	0.54679800
N	-0.16011100	0.34083900	0.61938200
H	-1.73372100	-0.05351000	-0.79800000
H	2.48274000	-0.32711700	-1.20210700
H	1.85825700	0.39220600	1.40960400
N	0.31869600	-0.25876900	-1.40699500
C	0.17781200	-0.60146400	-2.82814100
C	0.51282800	0.58366900	-3.73113400
H	-0.85391300	-0.93615200	-2.96524900
H	0.85649900	-1.43667600	-3.02262300
H	0.35244800	0.29856200	-4.77729700
H	-0.11091500	1.44859000	-3.48608900
H	1.56625700	0.86115100	-3.61735000
C	-0.91986300	0.67519700	1.82128200
H	-0.22661500	1.10108200	2.54975200
H	-1.67558100	1.41229700	1.54242900
H	-1.39460700	-0.23355000	2.19791300
C	-3.42439300	3.54071900	-2.36561600
H	-3.25199700	4.62368600	-2.38763200
H	-3.35200300	3.18782100	-3.40091600
H	-4.43115000	3.36210600	-1.97351300
C	2.61528800	2.85787600	0.73520600
C	1.79755600	3.18118500	-1.30469000
C	3.02846700	2.60072800	-1.42693300
N	3.51998900	2.40908500	-0.14532400
H	2.62367300	2.72948600	1.81830500
H	1.03149600	3.42042000	-2.02561500
H	3.57425800	2.25438100	-2.29020900
N	1.56664500	3.33423100	0.05160900
C	0.32332100	3.81694000	0.67966500
C	0.05360700	5.28011000	0.34976100
H	0.44815500	3.65467700	1.75253200
H	-0.48736000	3.18851500	0.30767500
H	-0.87013600	5.59395000	0.84746000
H	0.86953000	5.92712100	0.69155400
H	-0.08426800	5.41615800	-0.72743700
C	4.75618900	1.70297400	0.20719700
H	5.60358200	2.39365000	0.16521700
H	4.63418400	1.30639000	1.21793000
H	4.89411500	0.88446100	-0.50373100
C	2.49634300	1.10575500	3.76982000
O	3.18407700	0.41744000	2.95192400
O	1.75458500	2.07983900	3.44844000
C	2.52919100	0.69758600	5.24572200
H	2.43993500	1.57580600	5.89223100
H	1.66732500	0.04850600	5.45379800
H	3.44109700	0.14147500	5.48210900

C	2.31793000	-2.95167800	-0.11011900
C	1.78184000	-2.48849000	1.99138500
C	0.64530300	-2.91008100	1.35973100
N	1.00954700	-3.19865200	0.05310900
H	2.82635700	-2.94198900	-1.08213500
H	1.94946900	-2.10415100	2.98506300
H	-0.39162600	-2.94923100	1.67015400
N	2.80723900	-2.52638200	1.05991000
C	4.19341500	-2.06799300	1.26716600
C	5.08536800	-3.19240000	1.78644100
H	4.13523600	-1.23499900	1.97174300
H	4.53458400	-1.69043000	0.29840000
H	6.10204400	-2.81120100	1.93188900
H	4.72532400	-3.57395300	2.74867100
H	5.13091600	-4.02536700	1.07589500
C	0.12262800	-3.64769900	-1.02035000
H	0.69990800	-3.66769500	-1.94550900
H	-0.24784000	-4.65089300	-0.79410100
H	-0.71830200	-2.96012200	-1.11977100
C	3.95974000	-1.45944300	-2.88309200
O	3.07686100	-2.36951200	-2.85070900
O	4.28781300	-0.72789700	-1.89839100
C	4.64193900	-1.19478800	-4.22793200
H	4.79813500	-2.13088100	-4.77275600
H	5.59171700	-0.66949400	-4.09301300
H	3.98293700	-0.56524500	-4.84109100
C	-4.68387500	0.31056400	-0.06227600
C	-5.90020300	-1.35446400	0.75437800
C	-6.05335700	-1.34739200	-0.59994800
N	-5.30099700	-0.29467800	-1.08554100
H	-3.98432800	1.15621200	-0.15130300
H	-6.30160600	-2.01332300	1.50648500
H	-6.60425800	-2.00411300	-1.25416400
N	-5.05066000	-0.30628800	1.06731600
C	-4.48238000	-0.01811900	2.39415900
C	-5.55686100	0.14053400	3.46342200
H	-3.90465100	0.90085900	2.28126600
H	-3.78414400	-0.83249900	2.60990100
H	-5.07907000	0.38721200	4.41710900
H	-6.25565400	0.94490600	3.20985100
H	-6.12759200	-0.78267300	3.61100700
C	-4.97548700	-0.06242500	-2.49127300
H	-5.88243800	-0.15607800	-3.09239100
H	-4.57033700	0.94413400	-2.59549800
H	-4.22692500	-0.80189800	-2.78676700
C	-2.94526700	-2.57127900	0.03119900
O	-2.92850800	-1.94499300	-1.07028800
O	-2.46554000	-2.15937400	1.12790100
C	-3.56419500	-3.97914300	0.02709900
H	-2.76166900	-4.71929700	-0.09109000
H	-4.06186600	-4.19203600	0.97863900
H	-4.26223700	-4.10669200	-0.80554000

5 a)

1-Ethyl-3-methylimidazolium acetate <u>Pentamer</u> with <u>dispersion</u> interactions:

C	2.01840000	-2.07369300	-2.82401500
O	2.84413100	-2.54905100	-1.98080300
O	0.80224000	-1.83870000	-2.61417400
C	2.22659900	-0.04718900	-0.25992900
C	0.37594900	1.18732800	-0.21903600
C	0.13230200	0.03134900	0.46315800
N	1.29539100	-0.72125300	0.41806700
H	3.25849100	-0.32798900	-0.39274900
H	-0.22260200	2.07446300	-0.40248600
H	-0.72788200	-0.32524900	1.01106100
N	1.69296100	1.11663900	-0.65502300
C	2.38868400	2.11686600	-1.47531200
C	1.95646300	2.04485200	-2.93776100
H	3.45617800	1.91598000	-1.35828800
H	2.15643300	3.10309400	-1.06505600
H	2.53267600	2.76964800	-3.52364800
H	2.11568600	1.04472100	-3.35307400
H	0.89692900	2.29639500	-3.03609700
C	1.47840200	-2.06070200	0.97428900
H	0.53825300	-2.36414800	1.43612500
H	1.74037100	-2.72717900	0.14924200
H	2.28441500	-2.02589000	1.71148400
C	2.58948400	-1.74650100	-4.21472000
H	1.82257800	-1.34523600	-4.88287000
H	3.40041300	-1.01142600	-4.12125600
H	3.02183200	-2.65004800	-4.66057400
C	-3.15053600	0.51359500	-1.36480200
C	-1.40163700	0.18581800	-2.69778400
C	-1.83983000	1.46734700	-2.87334300
N	-2.92633300	1.64993800	-2.03722700
H	-3.98922300	0.37498700	-0.68960500
H	-0.55142500	-0.36944400	-3.07834600
H	-1.45672800	2.27631800	-3.47321800
N	-2.23200200	-0.38732900	-1.74932900
C	-2.10877200	-1.76844100	-1.24642900
C	-3.10268700	-2.69820800	-1.93548900
H	-2.25587300	-1.74738000	-0.16384500
H	-1.07712900	-2.06765200	-1.44601000
H	-2.97540000	-3.71444700	-1.54885500
H	-4.13575300	-2.38636900	-1.74702300
H	-2.93366900	-2.71753200	-3.01744700
C	-3.65761100	2.90787800	-1.84361100
H	-4.02907800	3.25084600	-2.81327200
H	-4.49566300	2.70071200	-1.17479500
H	-2.95665500	3.63227300	-1.41779500
C	-1.24514700	-1.46026200	3.32113200
O	-1.32957600	-0.21623400	3.50916700
O	-1.56811100	-2.05755700	2.24441300
C	-0.66431900	-2.31844700	4.45034100
H	-0.81863200	-1.84507000	5.42425900
H	-1.09529300	-3.32467700	4.44366600
H	0.41802900	-2.42287400	4.29187300
C	1.25459000	3.40815800	1.88494000
C	1.07030200	1.57849500	3.13217300

C	2.38475100	1.71583900	2.78812200
N	2.47549900	2.86694500	2.01566800
H	1.02138700	4.22670700	1.19753500
H	0.52234000	0.80807900	3.65889400
H	3.23482200	1.06566500	2.94426400
N	0.38971200	2.64156500	2.55806100
C	-1.07270600	2.82704900	2.55955800
C	-1.54143100	3.60798100	3.78359700
H	-1.50327000	1.82310900	2.55536000
H	-1.31742300	3.33894200	1.62431700
H	-2.63039200	3.72084500	3.74569300
H	-1.28733400	3.07612300	4.70653700
H	-1.09337400	4.60769200	3.81468700
C	3.68942700	3.42087700	1.41527000
H	3.39542400	4.23413200	0.75000300
H	4.34461200	3.80596000	2.20177700
H	4.20664300	2.64348100	0.84883800
C	-0.04803100	4.81319400	-1.08204900
O	1.07860500	5.01021900	-0.53845500
O	-0.89788900	3.94326400	-0.71981500
C	-0.38681100	5.66550800	-2.31116000
H	0.03588000	6.67002000	-2.21596500
H	-1.46796600	5.72439600	-2.47028300
H	0.06597700	5.20179900	-3.19841400
C	5.54994400	-2.05147200	-1.28446800
C	7.10789500	-2.31242500	0.27035400
C	7.59039700	-1.44575500	-0.66515700
N	6.61056400	-1.31051000	-1.63308700
H	4.58026300	-2.14054200	-1.79769200
H	7.54110600	-2.67431300	1.18834200
H	8.52116600	-0.90360100	-0.71643300
N	5.84036100	-2.68248400	-0.14231600
C	4.87584100	-3.50035900	0.61271700
C	5.41573300	-4.89394100	0.91290300
H	3.97760000	-3.54408700	-0.00585500
H	4.64211300	-2.93133200	1.51681200
H	4.65537100	-5.46334900	1.45760000
H	5.65278700	-5.43426400	-0.00992000
H	6.31528900	-4.86095400	1.53791000
C	6.58969600	-0.29457700	-2.68151100
H	7.58274900	-0.21608800	-3.12902600
H	5.87050000	-0.59387400	-3.44556900
H	6.28520600	0.65152500	-2.22584500
C	5.34946400	0.30454300	1.12830600
O	5.19801600	0.71318400	-0.06603400
O	4.61022400	-0.52787000	1.72290500
C	6.51083700	0.91097300	1.93338900
H	6.12313500	1.71845200	2.56889800
H	6.95373400	0.16377200	2.59939200
H	7.27527000	1.33654500	1.27632200
C	-6.03355600	-1.91221600	0.81304000
C	-5.03908600	-3.90019200	0.86893300
C	-4.25650900	-3.02592900	1.56658200
N	-4.89878200	-1.79778100	1.51555900
H	-6.72338900	-1.09986400	0.52237500
H	-4.90653100	-4.94765500	0.64785600

H	-3.28052900	-3.11294600	2.03172300
N	-6.13835900	-3.18924900	0.40841600
C	-7.23889400	-3.70944000	-0.42126700
C	-7.32216000	-3.00592500	-1.77381600
H	-8.16974200	-3.58316700	0.13964200
H	-7.05696600	-4.78162200	-0.53347200
H	-8.14527800	-3.43714600	-2.35428900
H	-7.51461200	-1.93605100	-1.64187500
H	-6.39417100	-3.14009800	-2.33998700
C	-4.44721900	-0.56053400	2.17122700
H	-4.76712600	-0.57131400	3.21680700
H	-3.35792100	-0.52523900	2.13946100
H	-4.89924800	0.27787100	1.63693500
C	-6.99653900	1.07865600	-0.52096000
O	-5.76499100	1.12385800	-0.20398800
O	-7.74727700	0.07223200	-0.37900500
C	-7.59854400	2.34393400	-1.13842000
H	-7.19110000	2.48250900	-2.14850800
H	-8.68697500	2.27279000	-1.20539900
H	-7.31619200	3.22393900	-0.55003100

1 b)

1-Ethyl-3-methylimidazolium acetate <u>Monomer without dispersion</u> interactions:

C	2.76830700	-0.28759100	-0.01869800
O	1.82314500	-1.13354800	-0.17648600
O	2.60642300	0.95149700	0.14823400
C	-0.56975700	0.31407200	-0.10808700
C	-2.78588600	0.44473800	-0.25505200
C	-2.30157100	1.70224700	-0.04155500
N	-0.92247400	1.59731600	0.04725500
H	0.46133500	-0.13770800	-0.10270300
H	-3.79697400	0.09034700	-0.38516000
H	-2.81109700	2.64957700	0.04848900
N	-1.68959600	-0.40213400	-0.29459300
C	-1.69345900	-1.86553500	-0.48075400
C	-2.21528000	-2.60665400	0.74952800
H	-0.65848900	-2.14421200	-0.69448500
H	-2.30075500	-2.08314100	-1.36560400
H	-2.18996400	-3.68509600	0.55940600
H	-1.58662700	-2.40017300	1.62155300
H	-3.24866300	-2.32881400	0.98692200
C	0.03263500	2.69409400	0.26905300
H	-0.17884400	3.16497300	1.23307500
H	1.03970800	2.25680100	0.26358200
H	-0.07237600	3.42876700	-0.53399300
C	4.19390100	-0.84741100	-0.03757000
H	4.93233700	-0.05389300	0.10474600
H	4.30598500	-1.59998000	0.75237100
H	4.37902200	-1.35507800	-0.99200100

2 b)

1-Ethyl-3-methylimidazolium acetate <u>Dimer without dispersion</u> interactions:

C	1.57746000	-2.39029700	-0.60179300
O	0.75095100	-2.25611800	0.35612900
O	1.68149500	-1.60819800	-1.59049400
C	-1.57031800	-0.86531400	-0.80793900
C	-3.68468800	-0.20777400	-0.85475900
C	-3.09595000	0.17948400	-2.02206400
N	-1.78297600	-0.24893400	-1.97596400
H	-0.62283100	-1.29877700	-0.44962400
H	-4.68820300	-0.05858400	-0.48971700
H	-3.48865900	0.72848600	-2.86380900
N	-2.71785900	-0.86799100	-0.11790800
C	-2.84216300	-1.35640000	1.26672700
C	-4.01338800	-2.31946700	1.44534800
H	-2.92158600	-0.47003700	1.90371600
H	-1.89416700	-1.85003600	1.49274400
H	-4.03583800	-2.66674200	2.48410200
H	-4.97744800	-1.84065500	1.23928700
H	-3.91384100	-3.19485200	0.79417200
C	-0.73878700	0.05015000	-2.95891700
H	-0.54181700	1.12528800	-2.93179800
H	0.16006900	-0.50677400	-2.67517100
H	-1.08059000	-0.25389900	-3.95215000
C	2.49351600	-3.62244000	-0.56407700
H	3.40173000	-3.45935200	-1.15253600
H	2.74900200	-3.89178400	0.46609400
H	1.95651100	-4.47406400	-1.00268800
C	1.57031900	0.86531200	0.80794200
C	3.68469100	0.20777700	0.85474900
C	3.09595800	-0.17948800	2.02205400
N	1.78298300	0.24892700	1.97596200
H	0.62282900	1.29877500	0.44963300
H	4.68820400	0.05859100	0.48970300
H	3.48867200	-0.72849300	2.86379500
N	2.71785700	0.86799600	0.11790600
C	2.84215400	1.35641200	-1.26672700
C	4.01337900	2.31947700	-1.44535100
H	1.89415700	1.85004800	-1.49273500
H	2.92157300	0.47005100	-1.90372000
H	4.03582300	2.66675700	-2.48410500
H	3.91383800	3.19486000	-0.79417200
H	4.97744100	1.84066400	-1.23929900
C	0.73880200	-0.05016000	2.95892400
H	1.08061200	0.25389200	3.95215300
H	-0.16005700	0.50676200	2.67518400
H	0.54183400	-1.12529800	2.93180600
C	-1.57746300	2.39029400	0.60179800
O	-1.68150200	1.60820000	1.59050400
O	-0.75095400	2.25611200	-0.35612600
C	-2.49351700	3.62243700	0.56407500
H	-2.74901100	3.89177000	-0.46609800
H	-3.40172700	3.45935800	1.15254300
H	-1.95650800	4.47406600	1.00267100

3 b)

1-Ethyl-3-methylimidazolium acetate <u>Trimer without dispersion</u> interactions:

C	5.02899200	-1.70264500	-1.16295700
O	4.51343100	-1.74337400	-2.32044900
O	4.49492300	-1.17876600	-0.13696600
C	1.77529400	-0.90782800	-1.93881400
C	-0.15986700	-1.07027600	-0.84855600
C	-0.01144200	0.22827700	-1.24510500
N	1.19841700	0.30298400	-1.92558500
H	2.79711600	-1.15921900	-2.28254100
H	-0.93491000	-1.57053700	-0.27223500
H	-0.61194400	1.11365600	-1.05547400
N	0.96054100	-1.75647500	-1.29772600
C	1.26647100	-3.17679800	-1.04540200
C	1.75305900	-3.42303100	0.38163300
H	2.03254500	-3.46090000	-1.77059200
H	0.35411700	-3.74282100	-1.25545400
H	1.98809800	-4.48736600	0.49973300
H	2.65737700	-2.83890800	0.57855000
H	0.97497700	-3.16598500	1.10768400
C	1.73889100	1.49848700	-2.57880200
H	1.52753400	2.37349000	-1.95685600
H	2.81484000	1.36636000	-2.70683100
H	1.27200000	1.62649400	-3.56064300
C	6.42330000	-2.32701200	-1.00960000
H	6.70423100	-2.42837300	0.04280800
H	6.45841100	-3.30429800	-1.50368000
H	7.16025900	-1.68752800	-1.51265000
C	1.63681400	1.87667100	1.46366700
C	2.86413900	0.13651500	2.08960200
C	1.64539300	0.03759600	2.69711900
N	0.89473000	1.12891800	2.29139200
H	1.28801100	2.74520700	0.88937100
H	3.71157500	-0.53187000	2.05016200
H	1.23740300	-0.71824700	3.35003100
N	2.83726000	1.29464300	1.33351200
C	3.94915600	1.80991600	0.51054600
C	5.05549200	2.42583500	1.36645500
H	3.51481400	2.55430100	-0.16134300
H	4.31828800	0.95404100	-0.06343700
H	5.85127100	2.79649400	0.71089900
H	4.68349900	3.26592200	1.96412900
H	5.49652400	1.68171500	2.03813200
C	-0.49220600	1.42575600	2.67031000
H	-0.50865900	1.88920900	3.66195600
H	-0.90094600	2.10802300	1.92032800
H	-1.07601000	0.50003700	2.67607600
C	-0.54708000	3.96080900	-0.56448200
O	-1.34465100	3.02849100	-0.23036600
O	0.71549400	3.89688800	-0.49712100
C	-1.17093700	5.26338800	-1.08209100
H	-1.87699500	5.04763500	-1.89297100
H	-1.74175300	5.74151600	-0.27566400
H	-0.40521700	5.95886000	-1.43532200
C	-4.05723000	-0.12683400	0.02730200
C	-5.06689400	0.16905000	-1.92478900
C	-5.03199900	1.37260100	-1.28342500

N	-4.40834400	1.16286800	-0.06818800
H	-3.49938200	-0.57079800	0.87419100
H	-5.46519000	-0.10589500	-2.88929900
H	-5.37929000	2.34833200	-1.58616800
N	-4.45267600	-0.75169900	-1.09378100
C	-4.28940500	-2.19641500	-1.36107500
C	-5.59623700	-2.96576800	-1.17175800
H	-3.51458600	-2.55043400	-0.67180300
H	-3.91882400	-2.28874500	-2.38728800
H	-5.42006500	-4.02630100	-1.38153900
H	-5.95224700	-2.88121900	-0.13955800
H	-6.38693600	-2.61274500	-1.84434600
C	-4.09561800	2.19564200	0.92959800
H	-3.84980600	1.69337600	1.86649600
H	-3.23730600	2.78002200	0.57589600
H	-4.97911000	2.82277400	1.07301000
C	-2.24766200	-2.33748000	2.05735800
O	-2.14384000	-2.79890300	0.88222000
O	-2.55899000	-1.13663400	2.33908100
C	-2.01433600	-3.30061000	3.22547400
H	-1.67797500	-2.76582000	4.11911600
H	-1.29815300	-4.08198500	2.95365700
H	-2.96454700	-3.79283100	3.47164700

4 b)

1-Ethyl-3-methylimidazolium acetate <u>Tetramer without dispersion</u> interactions:

C	-2.73771300	2.78803800	-1.97716600
O	-3.37888300	2.07599400	-1.13171900
O	-1.70197900	3.45338100	-1.73846000
C	-0.79902700	-1.23380800	-0.69777900
C	1.31841000	-0.59971200	-0.95339200
C	0.78358900	0.11502800	0.07878000
N	-0.53223500	-0.29254800	0.21351400
H	-1.73005100	-1.80369000	-0.73918800
H	2.33153900	-0.64114800	-1.34985700
H	1.22687900	0.80928000	0.78566900
N	0.31306000	-1.43581300	-1.42150900
C	0.45963300	-2.40260900	-2.52154600
C	0.32058600	-1.74331200	-3.89438700
H	-0.30650000	-3.16917100	-2.37349600
H	1.44703000	-2.86317800	-2.41031900
H	0.44341600	-2.50126700	-4.67632100
H	-0.66295300	-1.27554100	-4.01606000
H	1.09046200	-0.97861900	-4.04101900
C	-1.48878700	0.22835500	1.19184700
H	-0.92356600	0.62289300	2.03878400
H	-2.08182300	1.02057800	0.72280700
H	-2.12717500	-0.59590200	1.51697000
C	-3.29669900	2.80413200	-3.41056200
H	-4.36772900	3.03979000	-3.39960600
H	-2.77067600	3.53128700	-4.03518400
H	-3.18792700	1.80789200	-3.85952400
C	2.82055200	3.11153000	-0.30162400
C	1.34285900	3.42615600	-1.93160700
C	2.50418500	2.97662400	-2.49184600
N	3.41163800	2.78844700	-1.46100700

H	3.25109400	3.00426500	0.70867700
H	0.36335000	3.64267500	-2.34080500
H	2.75788100	2.75614500	-3.51718200
N	1.56564800	3.50213700	-0.56822400
C	0.58260100	3.95132800	0.44139200
C	0.70415800	5.44881100	0.72441800
H	0.76616800	3.35701500	1.34070400
H	-0.40072800	3.70618300	0.02950200
H	-0.04641000	5.73423800	1.47032800
H	1.69292300	5.70454500	1.12170300
H	0.52072500	6.03880400	-0.18046200
C	4.77080800	2.25039200	-1.59578800
H	5.30633300	2.82435400	-2.35711600
H	5.27761700	2.36705900	-0.63632100
H	4.72951400	1.18898700	-1.86630900
C	2.73328200	2.28012100	3.16419700
O	1.78823000	1.55814500	2.71763100
O	3.69551800	2.73329800	2.47548600
C	2.72239800	2.64289600	4.65569600
H	3.68377800	2.37956500	5.11211000
H	2.60736900	3.72844800	4.76564000
H	1.90884800	2.14094400	5.18738400
C	2.99513300	-2.76704700	0.93773000
C	2.16504200	-1.66722700	2.67444600
C	1.35435700	-2.73882400	2.43157100
N	1.89393800	-3.41168300	1.34831300
H	3.53791900	-2.99063900	0.01331400
H	2.06467500	-0.83361300	3.35431100
H	0.41334200	-3.04627000	2.86253400
N	3.18206500	-1.70688100	1.73453800
C	4.27167400	-0.72104500	1.58313800
C	5.45893100	-1.03461800	2.49342500
H	3.84948200	0.26007000	1.81458300
H	4.55428100	-0.73725900	0.52584800
H	6.22573400	-0.26409200	2.35942000
H	5.16537000	-1.03485800	3.54918500
H	5.90192100	-2.00813900	2.25350900
C	1.34480900	-4.62296800	0.73156400
H	1.77140500	-4.72151800	-0.26837300
H	1.61698000	-5.49671400	1.33189300
H	0.25714700	-4.51918500	0.69185200
C	4.41518600	-2.10650000	-2.32398200
O	3.78074300	-3.11804600	-1.89693400
O	4.30306700	-0.92720900	-1.86477300
C	5.41855400	-2.33137500	-3.46226600
H	6.40666100	-2.53363700	-3.02765800
H	5.50884300	-1.43960900	-4.09088600
H	5.13685000	-3.19751300	-4.06849000
C	-5.32192100	0.19590300	-0.36524100
C	-6.55796400	-0.28586700	1.40975600
C	-6.64344900	-1.33186100	0.53811100
N	-5.87578700	-1.00570600	-0.56422500
H	-4.62445300	0.75001200	-1.00921700
H	-7.00427500	-0.13821500	2.38099100
H	-7.16237300	-2.27487600	0.61213700
N	-5.72770900	0.65602200	0.82816100

C	-5.34322000	1.96859900	1.38903400
C	-6.49596000	2.97095200	1.35381800
H	-4.49753500	2.31549800	0.78720800
H	-5.00108600	1.79140500	2.41389100
H	-6.15647500	3.92219500	1.77758500
H	-6.81938300	3.15580700	0.32417600
H	-7.35915400	2.62931700	1.93684000
C	-5.59335100	-1.89205200	-1.70023900
H	-6.52471200	-2.36920500	-2.01459000
H	-5.20349000	-1.28601200	-2.51969900
H	-4.84552000	-2.62445500	-1.37575400
C	-2.74726500	-3.64190700	0.84036100
O	-3.25603500	-2.91840200	-0.07768700
O	-1.57995000	-3.52564700	1.30146600
C	-3.65360900	-4.73701000	1.42754300
H	-4.03952000	-5.37699400	0.62467100
H	-3.11833700	-5.34982200	2.15809200
H	-4.52037700	-4.27478900	1.91770500

5 b)

1-Ethyl-3-methylimidazolium acetate <u>Pentamer without dispersion</u> interactions:

C	0.89974400	-3.72402600	-1.60095100
O	2.06710700	-3.49165400	-1.15210400
O	0.10867100	-2.88158800	-2.09848400
C	2.03225500	-0.22844400	0.37772000
C	0.65253000	1.31265700	-0.43153700
C	0.09031600	0.80089600	0.70262500
N	0.96797100	-0.15709100	1.18660700
H	2.91319200	-0.84439300	0.55375700
H	0.33862400	2.09649300	-1.11587400
H	-0.79629300	1.05750600	1.27082400
N	1.86442100	0.66017800	-0.60915000
C	2.82446900	0.88935800	-1.70527500
C	2.54339300	-0.00236200	-2.91620000
H	3.81394400	0.69825900	-1.27988400
H	2.75357800	1.94774000	-1.97456200
H	3.34575900	0.12978300	-3.65258800
H	2.47557700	-1.05839600	-2.63359400
H	1.60288000	0.27577300	-3.40171200
C	0.74911000	-0.98812100	2.37202200
H	0.04458400	-0.46685400	3.02361700
H	0.32925300	-1.95256800	2.07037000
H	1.70557400	-1.14840500	2.87414200
C	0.42241200	-5.18639200	-1.50274200
H	-0.55849800	-5.32239900	-1.96737900
H	1.14799900	-5.85080600	-1.98748100
H	0.36289800	-5.48765100	-0.44858000
C	-3.43576200	0.30375600	-2.73234800
C	-1.37688500	-0.45322300	-3.10527200
C	-1.38636600	0.88785700	-3.35795900
N	-2.67417900	1.33915500	-3.11634000
H	-4.48723900	0.32744200	-2.42824700
H	-0.58170900	-1.18948400	-3.06823800
H	-0.60212500	1.59312400	-3.59203100
N	-2.66306300	-0.79191000	-2.71740900
C	-3.11285600	-2.14731100	-2.34614400

C	-3.49617900	-2.97685900	-3.57155600
H	-3.96372000	-2.01937800	-1.67204800
H	-2.27619100	-2.61093000	-1.81705400
H	-3.82101300	-3.97177900	-3.24659000
H	-4.31935500	-2.51343000	-4.12687400
H	-2.63906800	-3.10361900	-4.24083200
C	-3.11139000	2.73158900	-3.25283300
H	-3.43840800	2.92085100	-4.27997900
H	-3.94247300	2.91101300	-2.56810800
H	-2.26108600	3.37466400	-3.00931400
C	-1.43230300	2.03119400	3.96243500
O	-0.78232400	2.99682900	3.48320300
O	-1.84294100	1.01542400	3.31009500
C	-1.75428100	2.05346700	5.46654300
H	-1.45674100	3.00093400	5.92424800
H	-2.82725000	1.89195400	5.62810500
H	-1.22662600	1.23232100	5.96903800
C	2.46446900	4.24421600	0.35999900
C	1.90990400	3.91297100	2.48469200
C	3.24267600	3.64906200	2.34755500
N	3.56869700	3.86114400	1.01690500
H	2.37684900	4.39240000	-0.72640000
H	1.22230300	3.80601200	3.31393300
H	3.97537000	3.30869300	3.06274900
N	1.44946100	4.28427000	1.23369300
C	0.05907600	4.66142600	0.90398000
C	-0.21411700	6.13594000	1.20136500
H	-0.57730300	4.01322500	1.51306500
H	-0.08410200	4.43568800	-0.15672200
H	-1.25395900	6.36659500	0.94367100
H	-0.07456600	6.35807800	2.26488400
H	0.43675400	6.79293900	0.61294300
C	4.88552800	3.64688600	0.40510800
H	4.81300800	3.90728300	-0.65233200
H	5.61727700	4.30080500	0.88867400
H	5.17895300	2.59539100	0.51218900
C	1.03376200	4.20094000	-3.10636400
O	2.16751500	4.29616700	-2.55263600
O	0.03125000	3.57759000	-2.63355300
C	0.85239000	4.87402400	-4.47421700
H	1.70659100	5.51027600	-4.72034600
H	-0.06903300	5.46764900	-4.48647100
H	0.75118300	4.10429500	-5.25070900
C	4.89191600	-3.19676400	-0.65007900
C	6.48138300	-4.05162000	0.62634800
C	7.05622700	-3.13340200	-0.20071400
N	6.04930100	-2.62312100	-1.00096700
H	3.89984300	-3.01338700	-1.06279800
H	6.91215800	-4.66869300	1.39877400
H	8.07771000	-2.79650100	-0.28524400
N	5.13422000	-4.08469400	0.31975700
C	4.08161300	-4.84257300	1.02099100
C	4.31943400	-6.35012700	0.96773800
H	3.14596100	-4.56959800	0.52356900
H	4.05416700	-4.46921900	2.04977200
H	3.50485800	-6.86026500	1.49334700

H	4.33290300	-6.70891300	-0.06699300
H	5.25962300	-6.64268300	1.45012100
C	6.18261200	-1.49449200	-1.92580200
H	7.15098500	-1.56086300	-2.42699900
H	5.38745600	-1.55670600	-2.67072600
H	6.09342600	-0.56634900	-1.35040100
C	5.28719100	-0.33408200	1.54421700
O	5.41873200	0.43175700	0.54007000
O	4.56376700	-1.37197900	1.58954200
C	6.04511900	0.03905200	2.82985500
H	5.33975000	0.47759600	3.54865100
H	6.46459300	-0.85561400	3.30281200
H	6.84032100	0.76465900	2.63304600
C	-5.99906900	-1.03502600	1.88922900
C	-4.92178400	-1.88950300	3.63361700
C	-4.27534700	-0.74267400	3.27145700
N	-4.96876100	-0.23291300	2.18621200
H	-6.69543300	-0.92410500	1.03653400
H	-4.71409000	-2.59877600	4.42001000
H	-3.37776000	-0.23851000	3.62995900
N	-5.99048600	-2.05534700	2.76309700
C	-6.97002000	-3.15825800	2.77558900
C	-7.01530900	-3.91858000	1.45084500
H	-7.95113400	-2.73455500	3.01309300
H	-6.68044600	-3.81311000	3.60217800
H	-7.74175800	-4.73523100	1.53109300
H	-7.32543700	-3.26192900	0.63136800
H	-6.03652800	-4.35069200	1.21410000
C	-4.65266700	1.01704500	1.47987800
H	-5.20305600	1.84185400	1.94276400
H	-3.58220000	1.20032000	1.58621700
H	-4.95769100	0.89865200	0.43676300
C	-7.19565400	-0.44955300	-1.38063300
O	-5.98754100	-0.04719200	-1.36530900
O	-7.78366600	-1.01142400	-0.41409800
C	-8.00472900	-0.20263300	-2.65931700
H	-7.38300000	-0.34038600	-3.55079100
H	-8.87848000	-0.85854100	-2.70504400
H	-8.35664500	0.83745800	-2.66127700

Structures of EMIm Glycinate in xyz-coordinates:

1 c)

1-Ethyl-3-methylimidazolium glycinate monomer with dispersion interactions:

N	-4.42415700	1.11458200	-0.14369400
H	-3.97352100	1.75774600	0.50649800
C	-3.87796400	-0.22423600	0.08572000
C	-2.34506300	-0.38071400	0.02535400
H	-4.20789300	-0.57977100	1.06999100
H	-4.31419700	-0.91844700	-0.64267100
O	-1.64721100	0.66643000	-0.17870800
H	-4.11591800	1.43364500	-1.06162100
O	-1.88372500	-1.54352500	0.18913700
C	0.99767200	-0.31188400	-0.13573800
C	3.18600000	0.07252100	-0.25832400
C	3.00235300	-1.26448700	-0.05275100
N	1.63432600	-1.47944500	0.02128500
H	-0.09719000	-0.10625300	-0.12893200
H	4.08697400	0.65437400	-0.37497800
H	3.71297100	-2.07101200	0.04094100
N	1.92356600	0.64226700	-0.31011700
C	1.58901800	2.06815900	-0.46800800
C	1.88224300	2.86239400	0.80197300
H	0.52468900	2.10187700	-0.71285800
H	2.15848000	2.44775400	-1.32211400
H	1.61521900	3.91265400	0.64494600
H	1.28804500	2.47977800	1.63762500
H	2.94297000	2.81690600	1.07351200
C	0.95350000	-2.76499400	0.23068800
H	1.28226900	-3.19266900	1.18132400
H	-0.12376100	-2.56024200	0.24879800
H	1.20564800	-3.44196500	-0.58969100

2 c)

1-Ethyl-3-methylimidazolium glycinate dimer with dispersion interactions:

N	-4.02124800	-2.66042000	-0.60838900
H	-3.15775100	-3.17739400	-0.78005300
C	-3.86208100	-1.99446700	0.69259500
C	-2.41321500	-1.50991300	0.89803300
H	-4.53245600	-1.13167600	0.76310000
H	-4.09301700	-2.65117400	1.54901100
O	-1.51152200	-2.12795100	0.25119600
H	-4.78621300	-3.33173300	-0.58250600
O	-2.23061800	-0.55832300	1.70326000
C	1.02301800	-0.98024400	1.23346000
C	3.22472300	-0.68372300	1.14919700
C	2.72708500	0.10947100	2.14023500
N	1.35898300	-0.09342500	2.17865500
H	0.01515900	-1.33721800	0.98477900
H	4.20994500	-0.73843000	0.71272200
H	3.21225300	0.82771500	2.78145300
N	2.14426100	-1.35929500	0.60669700
C	2.16902300	-2.23770500	-0.57225700
C	3.18627900	-3.36369600	-0.42713100
H	2.38055200	-1.59256100	-1.42957300

H	1.15440400	-2.62947700	-0.67464100
H	3.14427500	-4.00424200	-1.31461400
H	4.20698900	-2.97553100	-0.34609500
H	2.97492500	-3.98045800	0.45320800
C	0.40355700	0.62747300	3.01807000
H	0.50022500	1.69589100	2.80811000
H	-0.59965500	0.27732900	2.75754400
H	0.61959700	0.43088300	4.07192300
N	4.65809100	0.77768200	-1.30165800
H	4.17396100	0.36478100	-2.10045200
C	3.80610700	1.87469300	-0.81686000
C	2.31454800	1.49367900	-0.90558500
H	4.04779200	2.11126600	0.22429400
H	3.92565100	2.80633300	-1.39597000
O	1.52358400	2.07974100	-0.11172600
H	5.55368300	1.13208400	-1.63190800
O	1.99505800	0.63713700	-1.78005200
C	-1.12354200	1.09974200	-0.97773000
C	-3.33074700	1.16462600	-0.76862100
C	-3.02617600	0.20026400	-1.68341300
N	-1.64658300	0.18690600	-1.80386100
H	-0.06461300	1.32508500	-0.81267300
H	-4.27782200	1.46726400	-0.35318700
H	-3.64939900	-0.53487000	-2.16801100
N	-2.13218900	1.71415900	-0.34801400
C	-1.94506700	2.68454600	0.73654300
C	-2.64006600	4.01176200	0.44869300
H	-0.86537700	2.81913100	0.83501500
H	-2.32140900	2.21171700	1.64895600
H	-2.46392900	4.70378300	1.27916900
H	-2.24917200	4.46644600	-0.46752100
H	-3.72334500	3.88850000	0.34028400
C	-0.85789300	-0.77269100	-2.57596200
H	-1.22636800	-0.79725500	-3.60439100
H	0.18289200	-0.44227300	-2.54819900
H	-0.96630800	-1.75090200	-2.10083200

3 c)

1-Ethyl-3-methylimidazolium glycinate trimer with dispersion interactions:

N	4.31834900	-2.03894300	-1.76466400
H	3.88896700	-1.59769000	-2.57763500
C	5.26734200	-1.07598800	-1.17923400
C	4.58944200	0.28846100	-0.94603600
H	5.62718700	-1.45830700	-0.21924800
H	6.15242600	-0.89501900	-1.81237500
O	3.65733400	0.60866500	-1.75033400
H	4.80140800	-2.87737700	-2.08330900
O	5.00278300	0.98057400	0.01868500
C	2.17297000	2.39162400	0.09497700
C	0.30634300	3.46281500	0.67836100
C	1.05265000	3.26513700	1.80274700
N	2.20934500	2.59824600	1.41925800
H	2.91791500	1.86589900	-0.51412200
H	-0.65994300	3.92364300	0.50280500
H	0.86489200	3.52967600	2.83150400
N	1.02605100	2.90757500	-0.36781200

C	0.59534500	2.86552500	-1.77438500
C	0.51164300	4.26427400	-2.37804500
H	-0.37741700	2.36704200	-1.78123900
H	1.32748200	2.24721500	-2.29803400
H	0.19989100	4.18828900	-3.42562800
H	-0.22391400	4.87644300	-1.84636800
H	1.48403500	4.76717900	-2.34545100
C	3.30519500	2.18712500	2.30065900
H	2.92267800	1.48282300	3.04409200
H	4.07172000	1.71074700	1.68176400
H	3.71183500	3.06900800	2.80290800
N	-2.68819200	4.12819300	-0.38732600
H	-2.62493800	3.60632500	-1.26151600
C	-3.25774300	3.22523700	0.62349500
C	-2.58859900	1.83870200	0.56429100
H	-3.10787700	3.64493400	1.62338800
H	-4.34283100	3.06016700	0.50232500
O	-2.60636900	1.15279100	1.61851000
H	-3.29243400	4.93303900	-0.54271900
O	-2.09004000	1.48927100	-0.54961300
C	0.12064000	-0.26968800	0.65822800
C	1.87740200	-1.08452400	1.73544000
C	2.09969200	-1.24476400	0.40005400
N	0.99574800	-0.71896400	-0.24753800
H	-0.83386300	0.19650100	0.43419900
H	2.47512500	-1.36917200	2.58578600
H	2.91584800	-1.67904300	-0.16647700
N	0.64447700	-0.46625500	1.87561800
C	-0.04186300	-0.15829600	3.13840400
C	-0.20874200	-1.40677200	4.00228500
H	-1.00228700	0.27971100	2.85689500
H	0.54151200	0.61177500	3.65808200
H	-0.86137800	-1.17532100	4.85147900
H	-0.63887900	-2.21949800	3.41134700
H	0.75085700	-1.75329000	4.40267700
C	0.79289700	-0.71491300	-1.69701900
H	0.58858900	-1.74038000	-2.01220600
H	-0.07292000	-0.08705000	-1.90802400
H	1.69517200	-0.30899400	-2.15854000
N	1.87662300	-4.64804000	-0.90781100
H	1.25607600	-4.66174600	-1.71715400
C	1.03967000	-4.55152000	0.29012200
C	-0.19481500	-3.62955200	0.20031600
H	0.66282100	-5.55369600	0.54110600
H	1.65210500	-4.23626200	1.14037300
O	-0.61767100	-3.33188300	-0.96205100
H	2.44207100	-3.80471400	-1.00944600
O	-0.71871400	-3.24936700	1.28499200
C	-3.03842700	-1.79099200	-0.44583600
C	-4.70490900	-0.50894600	-1.16027800
C	-4.83033000	-0.65345200	0.19019800
N	-3.78952100	-1.46112500	0.60954900
H	-2.12733400	-2.40995800	-0.45796500
H	-5.29331300	0.05103500	-1.86867400
H	-5.53999600	-0.23608000	0.88632800
N	-3.58446400	-1.23092500	-1.53467500

C	-3.00361800	-1.33779400	-2.88128100
C	-2.72107700	0.03617800	-3.48498900
H	-3.69268600	-1.91630800	-3.50632700
H	-2.08810900	-1.92182100	-2.76202700
H	-2.14515200	-0.08412000	-4.40882400
H	-3.64808000	0.56423600	-3.73577600
H	-2.16420400	0.65471900	-2.77680100
C	-3.48568300	-1.82961300	1.99384600
H	-4.33051000	-2.38296400	2.41307900
H	-2.58725500	-2.45174600	1.97394000
H	-3.30532500	-0.90816400	2.55018600

4 c)

1-Ethyl-3-methylimidazolium glycinate <u>tetramer with dispersion</u> interactions:

N	-3.64709600	-3.12020900	3.19173000
H	-2.69754800	-2.86464200	3.46205300
C	-4.56873200	-2.16611300	3.82780400
C	-4.12475600	-0.71705300	3.55680800
H	-5.57594700	-2.30745300	3.42382000
H	-4.63720100	-2.28324100	4.92274500
O	-2.88762900	-0.51089100	3.38174800
H	-3.82008100	-4.06841900	3.52086900
O	-5.03271700	0.16179200	3.52984500
C	-3.71011600	2.04919300	1.78376200
C	-2.84646500	3.40035500	0.24315500
C	-4.20213000	3.38959700	0.08456400
N	-4.72030600	2.54099200	1.05337600
H	-3.84114300	1.34453900	2.60868200
H	-2.08313000	3.92438300	-0.31288600
H	-4.83000500	3.90758900	-0.62332400
N	-2.56638200	2.55126700	1.30523600
C	-1.23835900	2.22339600	1.85983300
C	-0.62697800	3.41260600	2.59123700
H	-0.60896300	1.92187900	1.02127400
H	-1.40384600	1.37179100	2.52475900
H	0.33661400	3.11469200	3.01914800
H	-0.44374200	4.23970000	1.89985300
H	-1.27310400	3.74849000	3.40952200
C	-6.12531500	2.19383400	1.27453500
H	-6.54636200	1.78309700	0.35262300
H	-6.16023100	1.44430200	2.06935900
H	-6.68421900	3.08673100	1.56684800
N	-1.30804400	4.61813500	-2.48190700
H	-0.62941500	5.17489300	-1.96218500
C	-0.71933300	3.29542400	-2.70873800
C	-0.03764700	2.77671500	-1.42689400
H	-1.49905800	2.58760400	-3.01167900
H	0.04845800	3.27874500	-3.50367100
O	0.07410300	1.51920100	-1.30846400
H	-1.49962400	5.09270500	-3.36168100
O	0.36138300	3.64276700	-0.60454400
C	-2.42809300	-0.10215600	-0.80852100
C	-4.46834300	-0.88511700	-0.42977700
C	-3.62352800	-1.48364500	0.45701200
N	-2.36395900	-0.96777600	0.20616900
H	-1.58279500	0.44708400	-1.21676300

H	-5.52931900	-1.00272500	-0.58143600
H	-3.77484200	-2.21041800	1.25160700
N	-3.70695800	-0.01526700	-1.20105000
C	-4.17920900	0.74346100	-2.36414400
C	-4.59182100	-0.17420200	-3.51275900
H	-3.35741200	1.40034900	-2.65706700
H	-5.00548200	1.38165800	-2.03264800
H	-4.83004200	0.43006900	-4.39539000
H	-3.77970400	-0.87203100	-3.73972900
H	-5.48277700	-0.75626700	-3.25199300
C	-1.14516800	-1.36203300	0.91071300
H	-0.94832800	-2.40883000	0.67314600
H	-0.32973600	-0.73759000	0.54467000
H	-1.31142600	-1.20826000	1.97894600
N	-3.98160700	-4.82273400	-1.17781800
H	-3.19697900	-5.45536300	-1.02279600
C	-3.61553000	-3.91011800	-2.26054400
C	-2.26763500	-3.17191200	-2.12238600
H	-3.57996300	-4.47798000	-3.20032400
H	-4.40840000	-3.16574200	-2.38838500
O	-1.42783700	-3.63754000	-1.29424100
H	-4.04975800	-4.29118100	-0.30978800
O	-2.08991200	-2.15609900	-2.85758400
C	1.05609900	-2.21510900	-1.80062900
C	3.12944100	-1.61959000	-1.23224100
C	2.70718800	-0.80224600	-2.24098700
N	1.42095400	-1.19229500	-2.58226500
H	0.08057900	-2.72530800	-1.77928100
H	4.04115800	-1.64308000	-0.63906000
H	3.19684400	0.02290300	-2.73366100
N	2.07846800	-2.48855300	-0.97452100
C	2.06252800	-3.56998600	0.02805000
C	2.22543000	-3.04484000	1.45144800
H	2.87462800	-4.25951600	-0.22202200
H	1.11221400	-4.09177300	-0.10939400
H	2.16989400	-3.88786900	2.14899800
H	3.19705600	-2.55671500	1.58046200
H	1.42064800	-2.34636900	1.70504200
C	0.57413700	-0.58932200	-3.61528500
H	1.05751800	-0.70751900	-4.58917500
H	-0.38992900	-1.10586000	-3.59366200
H	0.43542800	0.46375800	-3.36550200
C	4.65595400	1.28100000	0.47596000
C	4.55127500	3.33626100	-0.35672800
C	3.28131800	2.96724400	-0.01679100
N	3.37747800	1.68534000	0.50130100
H	5.00677000	0.27908300	0.76384900
H	4.92872900	4.24741500	-0.79388400
H	2.31440000	3.45046100	-0.12444000
N	5.38911000	2.27328300	-0.04854600
C	6.85408900	2.23545400	-0.21577700
C	7.55888800	3.08107400	0.84133600
H	7.07105400	2.59469600	-1.22698900
H	7.14274900	1.18365500	-0.12778500
H	8.64091400	3.03124100	0.68055300
H	7.25331000	4.13314500	0.80023900

H	7.34764600	2.69221400	1.84263100
C	2.25663900	0.88848500	1.00371100
H	1.46352500	0.89816700	0.25107900
H	2.61713100	-0.12174100	1.19215700
H	1.89145700	1.32801100	1.93595900
N	8.47451600	-3.13317000	1.43774400
H	8.85517000	-2.80551000	0.55039500
C	7.01876200	-3.02270100	1.38104100
C	6.43565200	-1.65773500	0.96387100
H	6.63186800	-3.77466600	0.68005000
H	6.59955900	-3.28552400	2.35993500
O	7.22116300	-0.73470800	0.63796900
H	8.82437600	-2.46361400	2.12221400
O	5.15871500	-1.57255400	0.97416300

5 c)

1-Ethyl-3-methylimidazolium glycinate pentamer with dispersion interactions:

N	4.43121427	-3.24887581	-2.46415334
H	3.51824435	-3.13667053	-2.91623371
C	5.48655228	-2.52231581	-3.18899308
C	5.39399042	-1.00717963	-2.94369450
H	6.46236425	-2.86535403	-2.83436571
H	5.45337044	-2.68606557	-4.28069163
O	4.23331269	-0.48503573	-2.98038680
H	4.62382232	-4.24918811	-2.48968599
O	6.46458353	-0.38398430	-2.71260898
C	4.69805222	1.84593690	-1.31640580
C	4.03795700	3.61024324	-0.12248970
C	5.24463496	3.19569177	0.35991757
N	5.63884327	2.10030042	-0.39531499
H	4.68298991	1.01822096	-2.04719360
H	3.40625082	4.42359808	0.20914773
H	5.85490721	3.58855215	1.15812082
N	3.71918793	2.75125927	-1.16409515
C	2.50339106	2.78614460	-1.99766123
C	2.37560362	4.10088432	-2.75869649
H	1.64964104	2.63570494	-1.33303297
H	2.58683742	1.93422002	-2.67645372
H	1.49667175	4.05182206	-3.41067339
H	2.23149064	4.93841173	-2.07044988
H	3.25600159	4.28283956	-3.38430484
C	6.87811996	1.33556120	-0.22831495
H	6.85355476	0.81377634	0.73255588
H	6.94175618	0.61811533	-1.05402598
H	7.72647934	2.02445433	-0.25343905
N	2.33508875	5.44721290	1.96495033
H	1.74618002	6.05166583	1.39145023
C	1.60392485	4.19727640	2.18541713
C	0.85169090	3.77750838	0.90307189
H	2.30226776	3.40152422	2.46542473
H	0.85060676	4.26262470	2.99160009
O	0.58575045	2.54598322	0.76827699
H	2.53579123	5.92585644	2.83997178
O	0.54921781	4.70233616	0.10332744
C	2.61024246	0.25337907	0.86941431
C	4.40662334	-1.01179385	1.17153525

C	3.81528219	-1.40354129	0.00512688
N	2.70622556	-0.59396171	-0.15925811
H	1.84172013	1.01281397	1.00042513
H	5.28842508	-1.38066084	1.66950704
H	4.08156518	-2.16046000	-0.73844011
N	3.64499193	0.02723831	1.69304869
C	3.90732946	0.75961837	2.93818229
C	4.04606871	-0.16692279	4.14186855
H	3.06354482	1.43868512	3.07408620
H	4.80624614	1.36866692	2.78631453
H	4.21736438	0.43771061	5.03978067
H	3.12823259	-0.75014358	4.26652713
H	4.89872435	-0.84600274	4.03184656
C	1.71215236	-0.74396100	-1.22204780
H	1.06262925	-1.58455547	-0.97248034
H	1.12573856	0.17456185	-1.26115846
H	2.24946814	-0.89938336	-2.15933220
N	2.40791137	-4.39566499	1.80575291
H	1.54286129	-4.69740752	1.35041332
C	2.11737321	-3.45104621	2.87379223
C	1.27105421	-2.20336924	2.53873427
H	1.59617487	-3.97936203	3.68532038
H	3.05856391	-3.10549231	3.31984500
O	0.76262727	-2.12018920	1.38454167
H	2.94057860	-3.92180718	1.07869681
O	1.12005831	-1.35495963	3.47076924
C	-1.27835662	-0.07333122	1.28723894
C	-3.25958241	0.81618241	0.79262864
C	-2.80515437	1.39736984	1.94117739
N	-1.57954681	0.82297176	2.23693246
H	-0.40354350	-0.74728224	1.28400598
H	-4.20583537	0.91332583	0.27071310
H	-3.24539499	2.15135700	2.57450512
N	-2.28765084	-0.09127241	0.40236934
C	-2.33830800	-0.97645653	-0.77709448
C	-1.92460620	-0.29196527	-2.07297885
H	-3.36638356	-1.33301625	-0.85138554
H	-1.68904100	-1.83119332	-0.57965805
H	-0.95706036	0.20466943	-1.96131660
H	-1.81855281	-1.06533699	-2.84027329
H	-2.68221530	0.42134370	-2.40535987
C	-0.70986768	1.17326039	3.36040306
H	-1.32250426	1.27429825	4.26048041
H	0.02155075	0.36621986	3.48768920
H	-0.20570721	2.11321025	3.13003757
C	-3.85489268	3.33367635	-1.14066919
C	-3.72757851	4.87864497	0.44902656
C	-2.47233948	4.70532482	-0.06019646
N	-2.58203100	3.74507243	-1.05096363
H	-4.25442407	2.52812877	-1.77959445
H	-4.09420594	5.53389095	1.22383353
H	-1.50374901	5.12560321	0.17951077
N	-4.57067971	4.01403253	-0.23191915
C	-6.02596354	3.86929841	-0.04394713
C	-6.81073105	4.68513691	-1.06810066
H	-6.24014141	4.19770865	0.97734403

H	-6.24649946	2.79950303	-0.12994522
H	-7.88353490	4.56926751	-0.88010197
H	-6.56265301	5.75135465	-1.01424616
H	-6.60568355	4.32698174	-2.08213524
C	-1.45972215	3.21293063	-1.82909925
H	-0.79354987	2.67084532	-1.15500172
H	-1.86167220	2.56381038	-2.60543319
H	-0.91012986	4.04549559	-2.26897122
N	-6.75449314	-1.78525905	-1.02530136
H	-7.14062245	-1.09251286	-0.38033762
C	-6.17261334	-1.02795687	-2.14620121
C	-5.68015030	0.35994233	-1.69425474
H	-5.32018797	-1.56856466	-2.57518811
H	-6.88306909	-0.86052392	-2.97030252
O	-6.03665824	0.75696867	-0.54421370
H	-7.52185358	-2.37295671	-1.34478621
O	-4.94881754	0.99134559	-2.50580186
N	1.42900762	-3.80936517	-3.01486536
H	1.12188522	-4.48154565	-3.71675745
C	1.35362435	-4.43147096	-1.68497868
C	-0.02844881	-4.37829719	-1.00782659
H	1.69546527	-5.46976434	-1.73354177
H	2.04869265	-3.91187299	-1.01287322
O	-0.86778062	-3.55510362	-1.47577911
H	0.71827281	-3.07536452	-3.03328159
O	-0.20153090	-5.13821391	-0.00922150
C	-2.51126200	-3.91700004	1.17455818
C	-4.46403257	-2.84288013	1.09139825
C	-3.85771762	-2.52436195	2.27227754
N	-2.64961294	-3.20716955	2.30538136
H	-1.61873406	-4.48809623	0.84841037
H	-5.38093659	-2.50780368	0.61553746
H	-4.16715746	-1.87513102	3.07610807
N	-3.61067928	-3.71298084	0.43305237
C	-3.82404252	-4.25943428	-0.92163879
C	-3.73303715	-5.78162823	-0.93555408
H	-3.04028821	-3.83635556	-1.55574273
H	-4.80685624	-3.89976077	-1.23429312
H	-3.93384199	-6.14304567	-1.94996452
H	-2.72623294	-6.10745097	-0.65618615
H	-4.46354764	-6.23460034	-0.25536848
C	-1.67369052	-3.17630236	3.39472594
H	-2.16864594	-3.45357614	4.32906163
H	-0.88817060	-3.89535049	3.16724208
H	-1.21996918	-2.18767249	3.48102923

1 d)

1-Ethyl-3-methylimidazolium glycinate <u>monomer without dispersion</u> interactions:

N	-4.42964700	1.19710100	-0.14345300
H	-3.98966400	1.81042100	0.54195400
C	-3.91909200	-0.16105200	0.05725500
C	-2.38780800	-0.35902100	0.02955900
H	-4.28357300	-0.53835500	1.02121800
H	-4.35470500	-0.82129000	-0.70287600

O	-1.65580000	0.67231000	-0.13436500
H	-4.08956600	1.54099100	-1.04098400
O	-1.96471600	-1.53816300	0.17685900
C	0.99948500	-0.31259200	-0.10576000
C	3.19398700	-0.00089300	-0.27718600
C	2.97007200	-1.33186300	-0.07709900
N	1.59896900	-1.50299200	0.02734500
H	-0.09315800	-0.07850200	-0.08298200
H	4.11347400	0.54775400	-0.41230700
H	3.65755200	-2.16095600	-0.00587200
N	1.95214900	0.61366800	-0.29314800
C	1.66540900	2.05156800	-0.45880500
C	2.05921700	2.86808500	0.77131100
H	0.59155100	2.12373300	-0.64868700
H	2.19891400	2.39242900	-1.35221200
H	1.81869400	3.92235700	0.59697800
H	1.50351800	2.53355900	1.65311900
H	3.13207700	2.79659000	0.98376500
C	0.88252000	-2.77015300	0.24297700
H	1.19728900	-3.20207800	1.19687100
H	-0.19059300	-2.54029100	0.25623000
H	1.11927900	-3.45845300	-0.57288400

2 d)

1-Ethyl-3-methylimidazolium glycinate dimer without dispersion interactions:

N	-4.69690900	0.02937500	-1.35707800
H	-4.20046300	-0.42033200	-2.12764700
C	-4.63718500	-0.89215900	-0.20899900
C	-3.25268800	-1.56569400	-0.08821400
H	-4.84039700	-0.34590500	0.71908200
H	-5.37813300	-1.70659900	-0.26708400
O	-2.49329000	-1.52603900	-1.10036200
H	-5.66061700	0.17746400	-1.65250800
O	-3.00040300	-2.12796200	1.01469400
C	-0.08537900	-2.44933300	0.85025900
C	2.10686100	-2.08914500	0.87719600
C	1.67111800	-2.15534600	2.16958300
N	0.30174700	-2.37636300	2.13138600
H	-1.12878500	-2.53634500	0.53746700
H	3.08466200	-1.87002000	0.44929300
H	2.20611900	-2.04699000	3.10060500
N	0.99161300	-2.27997500	0.07598000
C	0.96842300	-2.27667800	-1.40127000
C	1.58439000	-3.54924600	-1.98164100
H	1.51291000	-1.38037400	-1.71328600
H	-0.08359700	-2.17651300	-1.68080300
H	1.54437100	-3.50026000	-3.07566400
H	2.63440300	-3.65749100	-1.68772100
H	1.03658000	-4.44269100	-1.66083600
C	-0.61367200	-2.49346300	3.26913800
H	-0.44356600	-1.65986100	3.95570500
H	-1.63482400	-2.45293100	2.88230200
H	-0.44117600	-3.43912200	3.79151900
N	4.65218700	-0.80041400	-0.70837200
H	4.23626600	-0.83502100	-1.64019200
C	4.51096300	0.58452800	-0.22537700

C	3.13496400	1.18094300	-0.59479700
H	4.62119100	0.61268000	0.86462900
H	5.27069200	1.26794300	-0.63930200
O	2.80054200	2.23223300	0.02165800
H	5.63501600	-1.05380900	-0.79718300
O	2.46519500	0.59298300	-1.49321200
C	-0.07632300	2.37513500	-0.49740400
C	-1.96389700	2.81241200	0.57859400
C	-2.27272800	2.03563500	-0.50127400
N	-1.07769200	1.78273400	-1.15660800
H	0.99685400	2.30272600	-0.69904600
H	-2.59331100	3.21929600	1.35507200
H	-3.21013200	1.59099200	-0.83523000
N	-0.59007800	3.00860000	0.56637900
C	0.21783200	3.76770500	1.53714400
C	0.09347900	5.27877700	1.34183100
H	1.24963100	3.43215300	1.39991100
H	-0.11439900	3.47075200	2.53759200
H	0.70967300	5.79309300	2.08760900
H	0.44738900	5.57238600	0.34808700
H	-0.94080100	5.62198600	1.45954300
C	-0.91128600	0.97028400	-2.36885400
H	-1.31670000	1.51506000	-3.22673300
H	0.15805200	0.79474900	-2.50317800
H	-1.44856600	0.02943500	-2.22151100

3 d)

1-Ethyl-3-methylimidazolium glycinate <u>trimer without dispersion</u> interactions:

N	3.02622200	-3.97910300	-1.92957400
H	2.77040900	-3.32558600	-2.67063000
C	4.31995800	-3.53910100	-1.38303500
C	4.37367000	-2.00614600	-1.21612000
H	4.48695400	-4.00209400	-0.40403500
H	5.17939800	-3.81627800	-2.01673600
O	3.52423300	-1.31719600	-1.85060000
H	3.10295900	-4.90652500	-2.34406300
O	5.28994600	-1.55783100	-0.46611300
C	4.32034300	1.09497300	0.14709500
C	3.16130200	2.86112200	0.83759800
C	3.98913000	2.47397200	1.85243400
N	4.70018100	1.37032000	1.40285400
H	4.67646500	0.21956800	-0.41239200
H	2.40393400	3.64217000	0.77450300
H	4.12176300	2.87338200	2.84633000
N	3.38930100	1.98481500	-0.21307200
C	2.72189200	2.00351500	-1.53220900
C	3.28417600	3.10159200	-2.43463100
H	1.65416500	2.14602200	-1.34056400
H	2.88410200	1.00964600	-1.95860200
H	2.76055700	3.07784200	-3.39701100
H	3.14178000	4.09617500	-1.99660600
H	4.35286300	2.95292900	-2.62664700
C	5.68909000	0.58342500	2.14302400
H	5.27218800	0.29449800	3.11152600
H	5.91260600	-0.31081300	1.55639400
H	6.59797200	1.17272300	2.29593800

N	0.48300900	4.69943000	0.48275000
H	0.29480800	4.45915400	-0.49172100
C	-0.48512800	3.95427600	1.30428500
C	-0.78752200	2.55598000	0.71366700
H	-0.09366500	3.82218900	2.31982800
H	-1.45403000	4.47134800	1.40479100
O	-1.40774300	1.76019600	1.47471600
H	0.35853600	5.70458000	0.58977400
O	-0.41758300	2.33458600	-0.47453700
C	-0.32044300	-1.02580600	0.96168700
C	0.67369300	-2.89629700	1.60823600
C	0.93421300	-2.72504300	0.27935400
N	0.30491600	-1.55047700	-0.09627800
H	-0.87229800	-0.08375800	0.98817300
H	0.97940000	-3.67444800	2.29011100
H	1.53200400	-3.30332700	-0.42408500
N	-0.10429300	-1.82330800	2.01735600
C	-0.65105300	-1.59681100	3.36448400
C	-1.68506900	-2.65245200	3.75681800
H	-1.09407900	-0.59811200	3.34720100
H	0.19263800	-1.57822900	4.06439400
H	-2.09358400	-2.40614400	4.74420900
H	-2.49793400	-2.67776900	3.02311000
H	-1.23348900	-3.64939700	3.82300700
C	0.26154800	-0.99951700	-1.45634300
H	-0.51220800	-1.52903700	-2.01807500
H	0.00224500	0.05859100	-1.37916600
H	1.25158300	-1.12407800	-1.90077700
N	-3.08564500	-4.72136200	-1.57636400
H	-3.34430400	-4.10175500	-2.34419700
C	-3.55981200	-4.11629800	-0.32947800
C	-3.29046500	-2.60752800	-0.12597600
H	-4.64752300	-4.25831100	-0.26141200
H	-3.13549000	-4.66400000	0.51981800
O	-2.89203200	-1.93381800	-1.12670300
H	-2.06597500	-4.71865900	-1.57845200
O	-3.51736500	-2.14028100	1.02784600
C	-3.89242400	0.74784100	-0.41492700
C	-4.71148200	2.69940400	-1.07359400
C	-4.95006400	2.56765700	0.26345000
N	-4.44040800	1.34187000	0.64940800
H	-3.41926400	-0.24210600	-0.45491400
H	-4.95184900	3.49715300	-1.75889100
H	-5.43257300	3.23151900	0.96417100
N	-4.05438800	1.54937600	-1.47648600
C	-3.55953800	1.23369400	-2.82957800
C	-2.44775300	2.18593700	-3.26918500
H	-4.41624900	1.26788900	-3.51186100
H	-3.20634600	0.20063100	-2.78213800
H	-2.05531400	1.85595500	-4.23787700
H	-2.81971200	3.21031900	-3.39220000
H	-1.63642300	2.19625500	-2.53384300
C	-4.38836200	0.80533000	2.01430500
H	-5.35601700	0.97092700	2.49498200
H	-4.17342900	-0.26424800	1.93763700
H	-3.58487600	1.31473500	2.55181100

4 d)

1-Ethyl-3-methylimidazolium glycinate **<u>tetramer without dispersion</u>** **interactions:**

N	-4.55592600	-1.81859600	3.69898600
H	-3.63370700	-1.45886300	3.94554800
C	-5.52618700	-0.72650200	3.87721900
C	-5.03067800	0.59037400	3.24537600
H	-6.47724500	-1.00130800	3.40809300
H	-5.74667000	-0.50442900	4.93513800
O	-3.79762800	0.69559600	2.98709700
H	-4.76369600	-2.59088500	4.33051000
O	-5.91275200	1.47930000	3.05553200
C	-4.69792600	3.08248200	1.01492000
C	-3.73167100	3.76682200	-0.86555300
C	-5.08114700	3.72277100	-1.07236400
N	-5.66398200	3.29188700	0.11008400
H	-4.88931200	2.64901200	2.00556700
H	-2.92136800	4.00381700	-1.55337000
H	-5.66633500	3.94793900	-1.95088000
N	-3.51990700	3.36246900	0.44478000
C	-2.22283900	3.26537100	1.14920600
C	-1.83954500	4.58790900	1.81334500
H	-1.47261500	2.97275600	0.40932700
H	-2.34514800	2.46763300	1.88755100
H	-0.87469500	4.46863500	2.31856000
H	-1.73640400	5.39064100	1.07462200
H	-2.58017400	4.88964400	2.56245800
C	-7.08740600	3.06057600	0.37037700
H	-7.50846400	2.45488400	-0.43638000
H	-7.17086000	2.52309400	1.31822900
H	-7.61715500	4.01601300	0.42636500
N	-1.38683600	3.97756900	-3.17687300
H	-0.65281500	4.42308400	-2.62469700
C	-1.01339700	2.56405100	-3.33640500
C	-0.33370400	2.01036500	-2.06225000
H	-1.90653700	1.96313800	-3.54693800
H	-0.31198400	2.38967100	-4.16984100
O	-0.23505000	0.75260800	-1.97564300
H	-1.43682000	4.44902500	-4.07815100
O	0.08583100	2.85913000	-1.22523300
C	-2.55460200	-1.09389700	-1.12186400
C	-4.53387700	-1.99184900	-0.71404700
C	-3.91163900	-1.79766600	0.48486100
N	-2.68133500	-1.23218700	0.20036100
H	-1.70073700	-0.64255300	-1.62029700
H	-5.50244200	-2.40774800	-0.94341100
H	-4.22516000	-1.97956700	1.51234800
N	-3.67593400	-1.53792200	-1.70496700
C	-3.89807700	-1.61701400	-3.15700800
C	-4.00313500	-3.06045600	-3.65041000
H	-3.05075300	-1.10785500	-3.62337200
H	-4.80219800	-1.04204700	-3.38902400
H	-4.06662800	-3.06352900	-4.74482700
H	-3.12694200	-3.63210300	-3.32709200
H	-4.90403300	-3.54995600	-3.26298700
C	-1.64245300	-0.91469000	1.18674800

H	-1.16368900	-1.84901500	1.49334400
H	-0.91144400	-0.26249900	0.70483700
H	-2.12005800	-0.40408900	2.02664200
N	-2.56290400	-6.18227200	0.90721300
H	-1.63990000	-6.33784600	1.31203600
C	-2.38218100	-5.65861400	-0.44770800
C	-1.40032300	-4.47720800	-0.62883100
H	-2.02667900	-6.47331100	-1.09386900
H	-3.35855000	-5.36435000	-0.84993600
O	-0.63917400	-4.18753200	0.34521400
H	-2.97520800	-5.45394700	1.48986100
O	-1.40969400	-3.89912100	-1.75502000
C	1.78190200	-3.00746500	-0.79222400
C	3.87845800	-2.25458700	-0.75319900
C	3.31376800	-1.89796200	-1.94354300
N	2.01333800	-2.37576900	-1.94931800
H	0.82933800	-3.45882500	-0.46476300
H	4.83969400	-2.00666700	-0.30976100
H	3.71797500	-1.34190900	-2.77547600
N	2.90209600	-2.94728800	-0.05324300
C	3.05352600	-3.55642400	1.28331100
C	3.26787300	-2.51594400	2.38116900
H	3.90290300	-4.24500000	1.23048400
H	2.14706400	-4.14256800	1.45455200
H	3.39937000	-3.03196500	3.33905300
H	4.16058300	-1.91084600	2.18821000
H	2.39495600	-1.85935700	2.47076500
C	1.01815600	-2.18026900	-3.01254200
H	1.45058500	-2.50436000	-3.96336700
H	0.14222000	-2.78814700	-2.76308500
H	0.74134900	-1.12242400	-3.04986600
C	4.70211000	1.89331100	0.14643900
C	4.25587900	3.87124300	-0.75144000
C	3.06562100	3.23755800	-0.54216800
N	3.37103300	2.00924700	0.01911500
H	5.23300300	0.99932900	0.51299100
H	4.47302600	4.83863000	-1.17723700
H	2.03673500	3.49508400	-0.76570300
N	5.26174300	3.01990200	-0.31882700
C	6.71430900	3.29282300	-0.34628200
C	7.10738500	4.39288200	0.63841600
H	6.97092400	3.56913200	-1.37487500
H	7.20611300	2.34895900	-0.08736100
H	8.18933500	4.55299700	0.57935100
H	6.61053300	5.34629000	0.42229200
H	6.86716300	4.09596700	1.66458200
C	2.38981600	1.01284600	0.46372800
H	1.59160800	0.93315700	-0.27825100
H	2.90700500	0.06230500	0.58893300
H	1.96385600	1.32872800	1.42067600
N	9.41781700	-1.46403300	1.78988800
H	9.81759900	-1.07449700	0.93657300
C	7.99327400	-1.70525500	1.56575300
C	7.14146300	-0.51199000	1.07441800
H	7.88333800	-2.51875800	0.83587700
H	7.54615600	-2.07797600	2.49634600

O	7.70671000	0.59129000	0.87700800
H	9.51589000	-0.71564800	2.47535400
O	5.89640500	-0.75093500	0.90262600

5 d)

1-Ethyl-3-methylimidazolium glycinate <u>pentamer without dispersion</u> interactions:

N	4.87842880	-3.00307934	-2.16421023
H	3.92785385	-3.06796860	-2.54160319
C	5.75853482	-2.22370861	-3.04666273
C	5.42642706	-0.71985794	-3.08659394
H	6.79126692	-2.32662422	-2.69653569
H	5.74906348	-2.57223106	-4.09544474
O	4.26095743	-0.34428287	-2.77755835
H	5.21331619	-3.96540963	-2.12335115
O	6.37030453	0.04559952	-3.45988912
C	5.56870623	2.55022206	-2.35810602
C	4.86487344	4.24672437	-1.10079621
C	6.23006499	4.22882643	-1.06521135
N	6.64683249	3.16493410	-1.85277067
H	5.62765145	1.62632196	-2.96182995
H	4.14019540	4.88993718	-0.59901492
H	6.93018137	4.86563500	-0.54622730
N	4.47951923	3.18853568	-1.91207865
C	3.09941597	2.80491953	-2.27340997
C	2.59634459	3.57851032	-3.49214344
H	2.47060235	3.00129059	-1.40062218
H	3.13364746	1.72901888	-2.47023393
H	1.57457605	3.25875444	-3.72531448
H	2.58171525	4.65831159	-3.30590160
H	3.21999954	3.37867923	-4.37022370
C	8.02097012	2.72162631	-2.10097233
H	8.53889068	2.58975409	-1.14722803
H	7.97371331	1.76661096	-2.62994442
H	8.54924609	3.46360608	-2.70665902
N	2.60820190	5.84761574	0.66273143
H	1.76292056	5.88689827	0.08948687
C	2.32844150	4.90228054	1.75669459
C	1.31066355	3.81424033	1.32885268
H	3.25292795	4.40558918	2.07469024
H	1.90976307	5.39259585	2.65098414
O	1.26898236	2.78069655	2.04920962
H	2.78957133	6.78378486	1.01973096
O	0.60242694	4.07015776	0.31050035
C	2.75693379	0.21244939	1.37422333
C	4.66145516	-0.87686855	1.64901731
C	4.11379906	-1.29026377	0.46923005
N	2.92686768	-0.59250389	0.32470612
H	1.94256868	0.91114457	1.53517741
H	5.58013336	-1.16309820	2.13734635
H	4.46198364	-1.97587463	-0.30662963
N	3.80618278	0.07215650	2.19343231
C	3.93913965	0.74544443	3.49254882
C	3.62008091	-0.18916811	4.66054239
H	3.25336668	1.59700322	3.46746378
H	4.96166949	1.13362589	3.56061690
H	3.68193961	0.36864224	5.60217851

H	2.61228102	-0.60130825	4.55521918
H	4.32750465	-1.02414977	4.71227126
C	1.95662190	-0.78754759	-0.75530773
H	1.35472427	-1.67433675	-0.54341025
H	1.30902908	0.09102201	-0.79242278
H	2.51162536	-0.88840830	-1.69060978
N	1.91574673	-4.46940590	2.63145018
H	1.32771936	-4.83775267	1.88049391
C	1.06414940	-3.86675848	3.65718043
C	0.37069544	-2.53132720	3.29988771
H	0.30410308	-4.59289332	3.96393215
H	1.67222464	-3.67236406	4.55502256
O	0.96698942	-1.77130977	2.47255458
H	2.46977352	-3.72501559	2.20819943
O	-0.72350721	-2.26378553	3.87848488
C	-1.40486427	-0.14953469	1.80382825
C	-3.41958725	0.59997582	1.23427074
C	-3.04502662	1.20931559	2.39714592
N	-1.79223637	0.73018947	2.73283191
H	-0.47554841	-0.73183659	1.86266315
H	-4.33421406	0.67390982	0.65130413
H	-3.56358056	1.92314193	3.01811857
N	-2.37431230	-0.24111065	0.88139943
C	-2.32345978	-1.12145582	-0.30188505
C	-2.00325403	-0.37843356	-1.59620783
H	-3.29991682	-1.60572964	-0.37029999
H	-1.57624062	-1.89553587	-0.11393129
H	-1.07478753	0.19400514	-1.49508606
H	-1.85315417	-1.11922177	-2.38815775
H	-2.81793632	0.28878700	-1.89554892
C	-0.99315226	1.07910679	3.91187510
H	-1.65161772	1.55589835	4.64153872
H	-0.59321239	0.14839063	4.32284481
H	-0.19420748	1.76364427	3.61258773
C	-3.95472509	3.47883256	-0.95183526
C	-3.67126928	4.88623004	0.73934521
C	-2.45846280	4.71248851	0.13804191
N	-2.66376131	3.84036083	-0.91597412
H	-4.41377292	2.72713591	-1.61980615
H	-3.95579417	5.47348627	1.59897576
H	-1.46034083	5.05190089	0.38060265
N	-4.58757466	4.10674186	0.05052553
C	-6.02681889	3.96104681	0.34724197
C	-6.88529240	4.94004741	-0.45346188
H	-6.14045232	4.12681690	1.42317192
H	-6.28341998	2.91817934	0.12720929
H	-7.93795045	4.80104694	-0.18316573
H	-6.61371186	5.98242291	-0.24976073
H	-6.78696358	4.75617180	-1.52842535
C	-1.62541911	3.40694026	-1.85772541
H	-0.70416476	3.25082243	-1.29113377
H	-1.95728130	2.48997386	-2.34408024
H	-1.47132013	4.18605377	-2.61035838
N	-7.02233779	-1.60234095	-1.18816728
H	-7.19772823	-1.00798315	-0.37391866
C	-6.51305325	-0.70411330	-2.24235544

C	-5.85771950	0.56508397	-1.65492590
H	-5.76821344	-1.21625450	-2.86275241
H	-7.30431904	-0.35940345	-2.92633964
O	-6.08452655	0.82581324	-0.43465580
H	-7.91440820	-2.00807245	-1.46512204
O	-5.15094077	1.25057491	-2.44569368
N	1.82290605	-4.03567879	-2.65118823
H	1.57986838	-4.64232038	-3.43334878
C	1.52699871	-4.73022622	-1.38781481
C	0.08714821	-4.55837257	-0.85110330
H	1.74433703	-5.79888373	-1.48460637
H	2.20249616	-4.35681033	-0.60448338
O	-0.63026041	-3.65599224	-1.37466282
H	1.16715581	-3.25425696	-2.71707230
O	-0.24681985	-5.31916878	0.10597046
C	-2.99466002	-4.67116900	0.81111502
C	-4.99640748	-3.72338333	0.58072283
C	-4.72591282	-3.83064533	1.91514760
N	-3.48196157	-4.42805039	2.03639038
H	-1.97723618	-5.04158583	0.56957671
H	-5.81838715	-3.26020809	0.04193168
H	-5.29547624	-3.52437524	2.77901986
N	-3.90512905	-4.25592447	-0.08488464
C	-3.74762419	-4.36669908	-1.54924907
C	-4.15962539	-5.74352375	-2.07018702
H	-2.69463932	-4.14958505	-1.76219514
H	-4.37146577	-3.58064163	-1.98386675
H	-4.03958975	-5.76690019	-3.15913437
H	-3.52598135	-6.52913165	-1.64506101
H	-5.20639317	-5.96942277	-1.83579528
C	-2.76233242	-4.68027684	3.29354821
H	-3.48881782	-4.97556636	4.05432845
H	-2.06135341	-5.49970191	3.12713720
H	-2.20733888	-3.78588195	3.60657995

2 e)

1-Ethyl-3-methylimidazolium glycinate <u>dimer with dispersion</u> interactions (double H-bonded):

N	-1.05665900	1.09919900	1.64768200
H	-0.66519500	0.30781800	1.14071700
C	-0.04674000	2.13964200	1.81304900
C	0.72763400	2.47713100	0.52297100
H	-0.51275500	3.06471200	2.16376700
H	0.72069900	1.89517600	2.56123700
O	0.38272500	1.92048800	-0.55126900
H	-1.34530600	0.75130100	2.55392700
O	1.70087300	3.25291800	0.68399800
C	3.12073000	0.73799100	0.42457300
C	3.26823100	-1.26086300	-0.50107500
C	3.60863400	-0.33508200	-1.43436600
N	3.50566900	0.90582100	-0.83903800
H	2.90039400	1.55326000	1.09441300
H	3.10818000	-2.32032800	-0.57239100
H	3.88711100	-0.44776200	-2.46454700

N	2.96768000	-0.56633600	0.65427200
C	2.40282200	-1.13467200	1.89376100
C	3.11035500	-2.40739400	2.31777500
H	1.34714400	-1.33032800	1.70056500
H	2.49229700	-0.35283800	2.64611600
H	2.71179600	-2.72490300	3.28092900
H	2.90731800	-3.20243800	1.60354500
H	4.18613000	-2.26035600	2.42278600
C	3.66591700	2.20581400	-1.48738500
H	3.27808600	2.13193500	-2.49963700
H	3.06908500	2.92446700	-0.92296400
H	4.71815700	2.48525600	-1.51178100
N	-0.37481400	-0.49369400	-2.16060200
H	0.08705200	0.21209600	-1.58708600
C	0.21686500	-1.81512600	-1.95166900
C	0.29495800	-2.21758200	-0.46234100
H	-0.39031400	-2.57064700	-2.45964300
H	1.22693900	-1.91454300	-2.36084400
O	1.05407700	-3.16944500	-0.19455300
H	-0.27950800	-0.20885200	-3.12766600
O	-0.39256200	-1.54148400	0.35423500
C	-2.82902500	0.20004900	-0.48843900
C	-4.58977000	0.21835100	0.84647500
C	-4.40265500	1.48084000	0.38436700
N	-3.31174500	1.44160500	-0.45544500
H	-1.95301400	-0.12973800	-1.05394700
H	-5.32229400	-0.18539900	1.51888600
H	-4.93460500	2.39079200	0.58588100
N	-3.60409300	-0.56546800	0.28202200
C	-3.37015300	-1.99518700	0.54072500
C	-3.46368700	-2.82027600	-0.73167700
H	-4.11733800	-2.30043200	1.27074400
H	-2.37178100	-2.08425900	0.96583800
H	-3.27742300	-3.86770600	-0.49770700
H	-4.44674900	-2.73458000	-1.19608200
H	-2.70542800	-2.50028800	-1.44397100
C	-2.65896700	2.58685400	-1.08406200
H	-3.03258400	2.72213300	-2.09787500
H	-2.87830700	3.47229200	-0.49396500
H	-1.58184700	2.41350900	-1.07686500
